# OIP5‐AS1 Sponges miR‐223‐3p to Upregulate FoxO3 and Ameliorate Temporomandibular Joint Osteoarthritis by Inhibiting Chondrocyte Apoptosis

**DOI:** 10.1111/jcmm.71359

**Published:** 2026-09-11

**Authors:** Xuesong Xu, Shichong Qiao

**Affiliations:** ^1^ Department of Stomatology, Huadong Hospital Fudan University Shanghai China; ^2^ National Clinical Research Center for Oral Diseases Shanghai Key Laboratory of Stomatology & Shanghai Research Institute of Stomatology Shanghai China; ^3^ Department of Implant Dentistry, College of Stomatology, Shanghai Ninth People's Hospital Shanghai Jiao Tong University School of Medicine Shanghai China

**Keywords:** cartilage protection, FoxO3, molecular mechanism, rno‐miRNA‐223‐3p, temporomandibular joint osteoarthritis (TMJOA)

## Abstract

Apoptosis and functional loss of articular chondrocytes serve as initiating events in temporomandibular joint osteoarthritis (TMJOA), and FoxO3 represents a critical molecule for sustaining chondrocyte homeostasis. This study investigates the regulatory mechanism of the lncRNA‐OIP5‐AS1/hsa‐miRNA‐223‐3p/FoxO3 axis in TMJOA, aiming to identify potential therapeutic targets for this disease. Single‐cell database analysis first revealed markedly reduced FoxO3 expression in TMJOA‐derived chondrocytes. A rat TMJOA model was then constructed and assigned to control, model, and FoxO3 overexpression groups; micro‐CT and histological staining, including HE and Safranin O‐fast green staining, were applied to assess articular cartilage injury, while immunohistochemistry was used to detect cartilage‐associated proteins Col2a1, MMP‐13, Aggrecan, and ADAMTS‐5. Further in vitro experiments validated the chondroprotective function of FoxO3 as well as the binding interaction between FoxO3 and rno‐miRNA‐223‐3p. Database screening confirmed significant down‐regulation of FoxO3 in TMJOA cartilage. Animal experiments demonstrated that FoxO3 overexpression mitigated chondrocyte injury in rat TMJOA lesions, increased Col2a1 and Aggrecan levels, and suppressed MMP‐13 and ADAMTS‐5 expression. Cellular assays showed that FoxO3 overexpression enhanced chondrocyte proliferation and matrix synthesis and preserved chondrocyte function. Sequencing and cellular evidence indicated that rno‐miRNA‐223‐3p directly targets FoxO3 mRNA to repress its transcription and negatively modulate FoxO3 abundance, consequently aggravating chondrocyte apoptosis. Collectively, rno‐miRNA‐223‐3p suppresses FoxO3 activity to facilitate TMJOA pathogenesis, and the lncRNA‐OIP5‐AS1/rno‐miRNA‐223‐3p/FoxO3 regulatory cascade may act as a promising molecular target for TMJOA clinical intervention.

## 1 Introduction

Temporomandibular joint osteoarthritis (TMJOA) represents a prevalent degenerative joint disorder marked by articular cartilage breakdown and aberrant subchondral bone remodelling. This pathological condition severely impairs masticatory function and lowers the life quality of affected patients [1]. Currently, the treatment of TMJOA mainly focuses on symptomatic relief, and there is a lack of targeted therapy for its etiology [2]. Thus, it is urgently needed to clarify the pathogenesis of TMJOA and explore potential therapeutic targets. Local inflammatory factors accelerate chondrocyte senescence and apoptosis, and regulate chondrocyte proliferation and apoptosis, which are important mechanisms driving disease progression [3]. As a transcription factor within the forkhead box family, FoxO3 exerts multifaceted regulatory effects on cell proliferation, apoptosis, inflammatory cascades and tissue repair [4]. Cumulative evidence indicates that FoxO3 confers protective effects on cartilage during osteoarthritis pathogenesis; nevertheless, its precise molecular mechanism has not been thoroughly clarified. Using single‐cell transcriptomic datasets, our research focused on FoxO3 and identified its tight correlation with inflammatory responses in chondrocytes [5]. To explore its underlying mechanism, we designed relevant experiments to verify the critical role of FoxO3 in inflammation‐driven chondrocyte apoptosis, aiming to provide a potential target for the treatment of TMJOA.

## 2 Materials and Methods [6]

### 2.1 Ethical Statement

All animal experiments in this research were performed in strict compliance with official ethical principles and institutional laboratory animal guidelines. The entire experimental protocol was formally authorized by the Institutional Animal Care and Use Committee (IACUC) of Huadong Hospital. All experimental animals were raised and maintained under standardized humane housing conditions throughout the study, and every measure was taken to alleviate potential pain and distress. Upon completion of the experimental procedures, all rats were sacrificed via humane euthanasia following ether anaesthesia.

### 2.2 RNA‐Seq Dataset and Sample Selection

Bulk RNA‐seq datasets accessible to the public were retrieved from the Gene Expression Omnibus (GEO) repository under accession number GSE232867.which provides transcriptomic profiles from a post‐traumatic temporomandibular joint osteoarthritis (TMJ‐OA) rabbit model ( *Oryctolagus cuniculus* ). Based on the sample annotations provided in the original dataset, six samples were included in this analysis: three control samples (GSM7385136, GSM7385138) and three OA samples (GSM7385142, GSM7385144). Differential expression analysis between OA and control specimens was implemented based on the limma package. A design matrix specifying the OA versus control contrast was applied, followed by empirical Bayes moderation of variance estimates. Genes with an absolute log_2_FC > 0.5 and nominal *p* < 0.05 were defined as differentially expressed and were used for subsequent enrichment and network analyses. Functional enrichment analysis was performed to explore the biological pathways enriched in differentially expressed genes. KEGG over‐representation analysis (ORA) was performed using rabbit Entrez Gene IDs via the clusterProfiler package, with enriched pathways defined as those with adjusted *p* < 0.05 [7].

Gene set enrichment analysis (GSEA) was carried out based on ranked moderated t‐statistics, enabling the evaluation of coordinated pathway changes without relying on fixed statistical cutoffs. Normalized enrichment scores and false discovery rate values were applied to identify significantly enriched biological pathways.

Enriched KEGG pathways were further visualized using Sankey flow diagrams and bubble plots to map pathway–gene relationships. All analyses and visualizations were implemented in R.

A high‐confidence protein–protein interaction (PPI) network was constructed to investigate potential intermolecular associations among differentially expressed genes. Gene sets were uploaded to the STRING database (https://string‐db.org), with the species specified as *Oryctolagus cuniculus* and the minimal interaction confidence score set at 0.90. The exported interaction dataset was subsequently imported into Cytoscape (version 3.10.1) for network visualization and topological characterization. Information obtained from differential expression analysis was integrated as node attributes to facilitate network graphical presentation.

### 2.3 Construction of Rat TMJOA Model

Specific‐pathogen‐free (SPF)‐grade housing conditions were applied for 8‐week‐old Sprague–Dawley rats, which were purchased from Beijing Vital River Laboratory Animal Technology Co. Ltd. (batch number: 101) (after 14 days post‐injection of AAV5‐CAG‐rFoxO3‐IRES‐EGFP). All rats were kept in a standardized laboratory environment with a 12‐h light–dark cycle, constant humidity ranging from 60% to 65%, and a stable ambient temperature of 22°C–25°C. Standard feeding conditions were provided throughout the housing period, allowing rodents ad libitum access to regular chow and sterile drinking water. Prior to formal experimentation, all animals underwent a one‐week adaptive feeding period to acclimate to the laboratory environment. General physical conditions and health status of the rats were routinely examined to ensure experimental eligibility. All animal handling procedures and experimental schemes were strictly reviewed and officially approved by the Institutional Animal Care and Use Committee.

A total of 18 Sprague–Dawley rats with body weights ranging from 180 to 200 g were randomly assigned to 3 experimental groups, including 6 rats allocated to the sham control group, two experimental groups (6 rats injected by AAV5‐CAG‐rFoxO3‐IRES‐EGFP, 6 rats injected by AAV5‐CAG‐NC‐IRES‐EGFP). The complete plasmid sequence of AAV5 vector for rat FoxO3 overexpression is constructed based on the pAAV2/5 backbone, containing all key functional elements. The sequence is oriented from 5′ to 3′, which can be directly used for plasmid synthesis and AAV5 virus packaging (Table S2). Dose: 1 × 10^11^ vg per TMJ, Volume: 15 μL per TMJ, Injection: Intra‐temporomandibular joint, Timing: TMJ.

OA Model establishment at 14 days post‐injection to construct the TMJOA model. Deep anaesthesia was induced with 4% isoflurane (1 L/min, R510‐22‐10, RWD Life Science, Shenzhen, China) in an O_2_ mixture to ensure complete unconsciousness, minimizing pain and stress. Tracheal intubation was performed on rats with a 20G catheter, followed by mechanical ventilation using a Harvard MiniVent Model 845 ventilator (Harvard Apparatus, USA). Animals were continuously anaesthetised with 2.0% isoflurane mixed with pure oxygen at a gas flow rate of 1.0 L/min. The ventilation parameters were configured with a tidal volume of 150 μL and a respiratory frequency of 150 breaths per minute.

Rats were secured on a surgical table for intra‐articular injections. Monosodium iodoacetate (MIA, I2512, Sigma‐Aldrich) was dissolved in sterile PBS to prepare a 0.5 mg/50 μL solution. A 27‐gauge, 0.5‐in. Needle was used to inject 50 μL of the MIA solution into the upper compartment of both TMJs of each rat, delivering a dose of 0.5 mg MIA per rat. The control group received a 50 μL injection of sterile PBS. Rats were then housed for 1 week to induce the TMJOA model.

4 weeks post‐treatment, all experimental rats were humanely euthanized. Bilateral temporomandibular joint tissues and major visceral organs, including the heart, liver, spleen, lung, and kidney, were surgically harvested. The collected specimens were processed via two preservation methods: some were fixed in formalin and embedded in paraffin for histological staining, while the remaining samples were immediately snap‐frozen in liquid nitrogen and stored at −80°C to support subsequent biochemical and histological detection.

### 2.4 Micro‐Computed Tomography (Micro‐CT) Observations

Following the intervention, all rats were euthanized, and temporomandibular joint (TMJ) tissues were precisely dissected. The isolated condylar specimens were fixed in 4% paraformaldehyde (PFA) for a 48‐h duration. To observe and record the macroscopic morphological characteristics of TMJs, a micro‐CT scanner (μCT50, Scanco Medical, Switzerland) was utilized for sample scanning. The scanning parameters were set at 70 kV and 114 μA, yielding an effective pixel resolution of 6 μm. Sagittal and top‐view three‐dimensional reconstructions of condylar bone structures were generated via Mimics software, and bone structural parameters were further analysed with CT vox software. Two core trabecular bone indexes were quantitatively measured in this study, including the bone volume/total volume ratio (BV/TV, %) and trabecular separation (Tb. Sp, mm) [8, 9].

### 2.5 Immunohistochemistry Staining

Prior to tissue sectioning, paraffin‐embedded samples were cooled on ice or stored at 4°C. After overnight air‐drying, the sliced sections were thermally baked in a 60°C oven for 20 min. For deparaffinization, sections were submerged in xylene (534,056, Sigma‐Aldrich, USA) for 10 min, followed by a second round of xylene immersion for an additional 10 min after solution renewal. The sections were subsequently dewaxed and rehydrated through two consecutive anhydrous ethanol (1,012,772, Sigma‐Aldrich, USA) treatments, each lasting 5 min. Gradient rehydration was further performed in 95% and 70% ethanol solutions for 10 min per concentration, and all sections were rinsed with distilled water for 5 min. For antigen retrieval, specimens were microwaved in citrate buffer (pH 6.0, C2488, Sigma‐Aldrich, USA) for 8 min and then cooled naturally to room temperature. After retrieval, sections were washed 3 times with PBS (pH 7.2–7.6, P5493, Sigma‐Aldrich, USA), with each wash lasting 3 min. To eliminate endogenous peroxidase activity, tissue slides were incubated in 3% hydrogen peroxide (88,597, Sigma‐Aldrich, USA) at room temperature for 10 min, followed by three successive 3‐min PBS rinses. Non‐specific antigen binding was blocked via 20 min incubation with normal goat serum (E510009, Sangon Biotech, Shanghai, China) at room temperature.

Upon completion of blocking, sections were incubated overnight at 4°C with primary antibodies targeting Col2a1 (sc‐518,017, Santa Cruz, California, USA), MMP‐13 (PME‐M100031, DIMA, Wuhan, China), Aggrecan (ab216965, Abcam, Cambridge, UK), and ADAMTS‐5 (ab41037, Abcam, Cambridge, UK). After repeated PBS washing, slides were treated with goat anti‐rabbit IgG secondary antibody (ab6721, Abcam, Cambridge, UK) for 30 min. Subsequently, the Streptavidin‐Biotin Complex (SABC, P0603, Beyotime, Shanghai, China) was added, and sections were incubated at 37°C for 30 min. Visualization of positive staining was conducted using a DAB Staining Kit (P0203, Beyotime, China); chromogen solution was applied to each section for 6 min, followed by brief haematoxylin counterstaining for 30 s. For final mounting, sections underwent gradient dehydration in 70%, 80%, 90%, 95% ethanol, and absolute ethanol (2 min for each solution), followed by two rounds of xylene clearing (5 min each). All processed slides were sealed with neutral resin. Microscopic observation and quantitative analysis were performed using an upright BX63 microscope (Olympus, Japan). 5 independent high‐power fields were randomly captured for each section, and the mean optical density (OD) values were quantified using Image‐Pro Plus 6.0 software version 6.0 [10].

### 2.6 Safranin O‐Fast Green Staining

Rat TMJ tissue sections were subjected to Safranin O‐Fast Green staining using a commercial staining kit (G1371, Solarbio Science & Technology, Beijing, China) in strict accordance with the manufacturer's standard operating instructions. Briefly, slides were first stained with Safranin O solution for 1 h, after which redundant staining solution was thoroughly washed away with distilled water. Gradient decolorization was performed using 50%–80% ethanol. Subsequently, sections were counterstained with Fast Green for 60 s and fully dehydrated with absolute ethanol. After xylene clearing for 5 min, all tissue slices were permanently mounted with neutral resin for subsequent observation.

Stained histological sections were visualized under a light microscope and photographed for further quantitative analysis. The positive area of Safranin O staining, which reflects the intracellular and extracellular aggrecan (AGG) abundance, was quantitatively measured via Image J software. Cartilage degenerative changes were assessed according to the Osteoarthritis Research Society International (OARSI) histological scoring system, with scores ranging from 0 (intact normal cartilage) to 6 (extensively damaged cartilage). All sections were evaluated independently by 2 blinded researchers, and consistent scoring results were recorded for statistical analysis.

### 2.7 Haematoxylin and Eosin (H&E) Staining

Haematoxylin and eosin (H&E) staining was carried out with a commercial staining kit (C0105S, Beyotime, Shanghai, China) to evaluate pathological morphological alterations in rat TMJ tissues as well as major visceral organs, including the heart, liver, spleen, lung, and kidney. All tissue specimens were initially fixed in 4% paraformaldehyde (P0099, Beyotime, Shanghai, China), followed by routine dehydration, transparency treatment, and paraffin embedding. The embedded tissue blocks were sliced into continuous 5 μm‐thick sections using a microtome. Subsequently, the sections underwent thermal baking, deparaffinization, and hydration prior to staining. For standard H&E staining, sections were first stained with haematoxylin solution and rinsed with distilled water to remove residual dye, then immersed in 95% ethanol for preliminary dehydration. Eosin staining and hydrochloric acid‐alcohol differentiation with 70% ethanol were performed sequentially. After completing gradient dehydration and xylene clearing, all sections were sealed with neutral gum. The general histological morphology and pathological changes of collected tissues were observed under an optical microscope. Ultimately, whole‐slide scanning and image acquisition were conducted using an Aperio ScanScope CS slide scanner (Leica Biosystems, Germany) for subsequent histological assessment.

For histological scoring of TMJOA, scores were assigned based on the number of neutrophils, synovial hyperplasia, inflammatory cell infiltration, and angiogenesis observed in each field. The synovial inflammation score was classified as no inflammation (0), mild inflammation (1), moderate inflammation (2), severe inflammation (3), and very severe inflammation (4). Cartilage erosion was scored based on cartilage surface integrity and the extent of subchondral bone erosion, with scores classified as no erosion (0), mild erosion (1), moderate erosion (2), severe erosion (3), and very severe erosion (4). Data were collected by averaging neutrophil counts, synovial inflammation scores, and cartilage erosion scores from five high‐power fields per rat. Each experimental group included at least six rats to ensure data reliability. All statistical analyses were performed using GraphPad Prism software. All quantitative data are expressed as the mean ± standard error of the mean (SEM). Intergroup differences were analysed via one‐way analysis of variance (ANOVA) followed by Tukey's multiple comparison test, with a statistical significance threshold defined as *p* < 0.05. This comprehensive analytical strategy, combining histological scoring and statistical verification, enables reliable quantitative evaluation of TMJOA tissue lesions and facilitates systematic assessment of the therapeutic effects of different interventions on inflammatory responses and tissue injury.

### 2.8 Isolation, Identification, and Cultivation of TMJ Chondrocytes

Primary chondrocytes were isolated from the TMJ cartilage tissues of 3 6‐week‐old Sprague–Dawley rats (body weight: 180–200 g; batch: 101, Beijing Vital River Laboratory Animal Technology Co. Ltd., Beijing, China) under sterile experimental conditions. The mandibular condylar cartilage was precisely dissected and transferred to a sterile culture dish. After being thoroughly rinsed two to three times with PBS solution, the cartilage tissues were cut into uniform 1 mm × 1 mm × 1 mm fragments using ophthalmic scissors. The minced tissue samples were digested with a mixed solution containing 0.2% Type II collagenase (17,101,015, Thermo Fisher, USA) and 0.25% trypsin (25,200,072, Thermo Fisher, USA) at 37°C for 3 h. The resulting cell suspension was filtered through a stainless‐steel cell strainer and centrifuged at 1000 rpm for 5 min. Chondrocytes at passages 3–6 were harvested and applied for all subsequent in vitro experiments.

An Alcian blue staining kit (G3660, Solarbio Technology Co. Ltd., Beijing, China) was utilized to identify the phenotypic characteristics of primary chondrocytes. Stained cellular samples were visualized and imaged using an inverted fluorescence microscope for morphological observation and cell identification.

### 2.9 Cell Viability Assay

The optical density value at 450 nm was detected via a microplate reader (BioTek, USA), and cell viability was subsequently quantified according to the corresponding calculation formula. The optical density value at 450 nm was detected via a microplate reader (BioTek, USA), and cell viability was subsequently quantified according to the following formula:

$$\text{Cell viability}\;\left(\%\right)=\left(\frac{{\mathrm{OD}}_{\text{sample}}-{\mathrm{OD}}_{\text{blank}}}{{\mathrm{OD}}_{\text{control}}-{\mathrm{OD}}_{\text{blank}}}\right)\times 100$$

OD sample denotes the absorbance value of drug‐incubated samples, OD control represents readings obtained from untreated chondrocytes, and OD blank corresponds to the blank control absorbance.

### 2.10 Lentivirus Transfection

Lentiviral vectors including Lv‐oe‐FoxO3 and negative control Lv‐NC were commercially obtained from OBiO Technology (Shanghai, China). Cell transduction was performed when chondrocytes reached 30%–50% confluence. Cell viability remained above 95% at 12 h post‐transduction. The culture medium was refreshed subsequently, and cell passaging was conducted 3 days later. An aliquot of cells was preserved for stock, whereas the rest were subjected to subsequent functional assays. Transduction efficiency was evaluated via fluorescence observation.

### 2.11 RNA Extraction From Sequencing Samples

Cell dissociation was performed with 0.25% trypsin–EDTA solution (25,200,056, Thermo Fisher, USA) for 3–5 min until cellular edges separated from the culture plate. Digestion was terminated by adding complete medium supplemented with 10% fetal bovine serum (FBS; 10100147C, Thermo Fisher, USA). The cell suspension was harvested via low‐speed centrifugation at 1000 rpm for 5 min, and the supernatant was discarded afterwards.

For total RNA isolation: Total RNA was purified utilizing Trizol reagent (A33254, Thermo Fisher, USA) in accordance with the manufacturer's official instructions. After RNA precipitation, the sediment was rinsed with 75% ethanol (E299585, Aladdin, Shanghai, China), air‐dried for 5–10 min, and resuspended in DEPC‐treated water.

For evaluation of RNA integrity and purity: The concentration and purity of extracted RNA were initially quantified with a Nanodrop ND‐1000 spectrophotometer (Thermo Fisher) to exclude protein and organic solvent contamination. The OD260/280 ratio served as the indicator for RNA purity. A Qubit RNA Assay Kit (Q33221, Thermo Fisher, USA) was further adopted to precisely determine RNA concentration. Only RNA specimens satisfying the following standards were applied to downstream experiments: RNA Integrity Number (RIN) ≥ 7.0 and a 28S: 18S rRNA ratio ≥ 1.5.

### 2.12 Transcriptome Sequencing Data Analysis

Quality evaluation of raw paired‐end sequencing reads was implemented via FastQC software (v0.11.8). Raw data preprocessing was completed with Cutadapt (v1.18) to trim Illumina sequencing adapters and poly (A) tails. Reads carrying over 5% ambiguous N bases were filtered out using a Perl script. The FASTX Toolkit (v0.0.13) was applied to preserve qualified sequences, requiring no less than 70% of bases to possess Phred quality scores exceeding 20. Damaged paired‐end sequences were restored with BBMap. Clean high‐quality reads were subsequently mapped to the rat reference genome utilizing HISAT2 (v0.7.12).

mRNA read count‐based differential expression analysis was carried out with the limma R package. Genes satisfying the thresholds of |log_2_ fold change (log_2_FC)| > 2 and *p* < 0.05 were identified as differentially expressed genes (DEGs). Functional enrichment profiling of DEGs was performed using the clusterProfiler R package, covering three major Gene Ontology (GO) branches: biological process (BP), molecular function (MF), and cellular component (CC). Bubble and circular plots were constructed to visualize GO enrichment outputs. Using identical |log_2_FC| cutoffs, Kyoto Encyclopedia of Genes and Genomes (KEGG) pathway enrichment was further conducted via clusterProfiler to screen candidate pathways. Bubble charts and circle plots were also adopted for graphical presentation of KEGG enrichment outcomes [11].

### 2.13 RT‐qPCR Analysis of Gene Expression

Total RNA was isolated from tissue and cellular specimens using a Trizol reagent kit (A33254, Thermo Fisher, USA). Purified RNA was reversely transcribed into complementary DNA (cDNA) with a Reverse Transcription Kit (RR047A, Takara, Japan) according to the standard protocols. Quantitative real‐time PCR reactions were configured with SYBR Premix Ex TaqTM II (DRR081, Takara, Japan) and conducted on the ABI7500 Real‐Time PCR System (Thermo Fisher, USA). The amplification procedure consisted of an initial pre‐denaturation step at 95°C for 30 s, followed by 40 cycles of denaturation at 95°C for 5 s and annealing at 60°C for 30 s. A final melting curve stage was subsequently performed at 95°C for 15 s, 60°C for 60 s, and 90°C for 15 s. Glyceraldehyde‐3‐phosphate dehydrogenase (GAPDH) was adopted as the endogenous reference gene for normalization. All RT‐qPCR assays were conducted in technical triplicates across three independent biological repetitions.

Relative gene expression levels between experimental and control groups were quantified via the 2 algorithms. Briefly, ΔCt was calculated as the difference between the Ct value of target genes and the reference gene, while ΔΔCt was defined as the ΔCt difference between experimental and control groups. The Ct value corresponds to the threshold cycle number at which the fluorescent signal exponentially exceeds the background level. Detailed primer sequences used in this study are listed in Table S1 [10].

### 2.14 Western Blot (WB)

Western blot assays were performed following standard laboratory procedures for protein expression detection. Briefly, total protein was extracted from tissue and cell samples using RIPA lysis buffer supplemented with 1% phenylmethanesulfonyl fluoride (PMSF; P0013B, Beyotime, Shanghai, China). The protein concentration of each lysate was accurately quantified with a BCA protein assay kit (P0011, Beyotime, Shanghai, China). Qualified protein samples were separated via sodium dodecyl sulfate–polyacrylamide gel electrophoresis (SDS‐PAGE) and electrotransferred onto polyvinylidene fluoride (PVDF) membranes.

After membrane blocking, immunoblotting was performed through sequential incubation with primary and secondary antibodies at optimized dilutions. The primary antibodies used in this study included rabbit anti‐FoxO3 (1:3000 dilution, ab219620, Abcam) and rabbit anti‐Col2a1. Rabbit anti‐GAPDH antibody (1:10,000 dilution, ab37168, Abcam, Cambridge, UK) was used as the internal loading control for protein normalization. Two types of HRP‐conjugated secondary antibodies were applied: goat anti‐rabbit IgG (1:5000, ab6721, Abcam, Cambridge, UK) and goat anti‐mouse IgG (1:5000, ab205719, Abcam, Cambridge, UK). Additionally, an HRP‐conjugated goat anti‐rabbit IgG antibody (1:10,000, AS1107, ASPEN) was utilised for enhanced signal detection.

Target protein bands were visualized using an enhanced chemiluminescence (ECL) substrate. The grey values of individual bands were analysed and quantified with ImageJ software (NIH, Bethesda, MD, USA) to evaluate relative protein expression levels.

### 2.15 Scratch Assay

A cell scratch wound healing assay was performed in 6‐well plates to evaluate cell migratory capacity. Briefly, uniform horizontal guidelines were marked on the bottom of each well at intervals of 0.5–1 cm, with a minimum of five reference lines per well. Chondrocytes were seeded at a density of 5 × 10 cells per well and cultured until reaching full confluence. A straight vertical wound was generated across the premarked lines using a sterile 200 μL pipette tip. Following scratching, the original culture medium was discarded, and each well was replenished with serum‐free medium to eliminate the influence of serum‐induced cell proliferation. Wound closure areas were visualized and photographed at 0 h and 24 h post‐scratching using a Leica DM500 inverted optical microscope. The wound gaps of different groups were statistically measured, and the wound healing rate was calculated via Image‐Pro Plus 6.0 software according to the following formula:

$$\text{Wound healing rate}=\frac{{\text{distance}}_{0\;h}-{\text{distance}}_{24\;h}}{{\text{distance}}_{0\;h}}$$

where distance0 h and distance24 h represent the distance between the edges of the scratch at 0 and 24 h, respectively.

### 2.16 Statistical Analysis

All quantitative data were derived from no fewer than three independent biological experiments and presented as the mean ± standard deviation (SD). For statistical comparison between 2 independent groups, the 2‐sample *t*‐test was adopted. One‐way analysis of variance (ANOVA) was utilized for multigroup statistical analysis, followed by Tukey's honestly significant difference (HSD) post hoc test to identify specific intergroup differences when overall statistical significance was observed. For datasets that failed normality testing or exhibited unequal variance, non‐parametric statistical analyses were performed, including the Mann–Whitney *U* test for two‐group comparisons and the Kruskal‐Wallis *H* test for multiple‐group comparisons. All statistical computations were implemented using GraphPad Prism 9.5.0 (GraphPad Software Inc.) and R 4.2.1 (R Foundation for Statistical Computing). A 2‐tailed *p*‐value < 0.05 was defined as the threshold of statistical significance for all analyses.

## 3 Results

### 3.1 Decreased Expression of FoxO3 in TMJOA Rabbit Cartilage Cells

**FIGURE 1:**
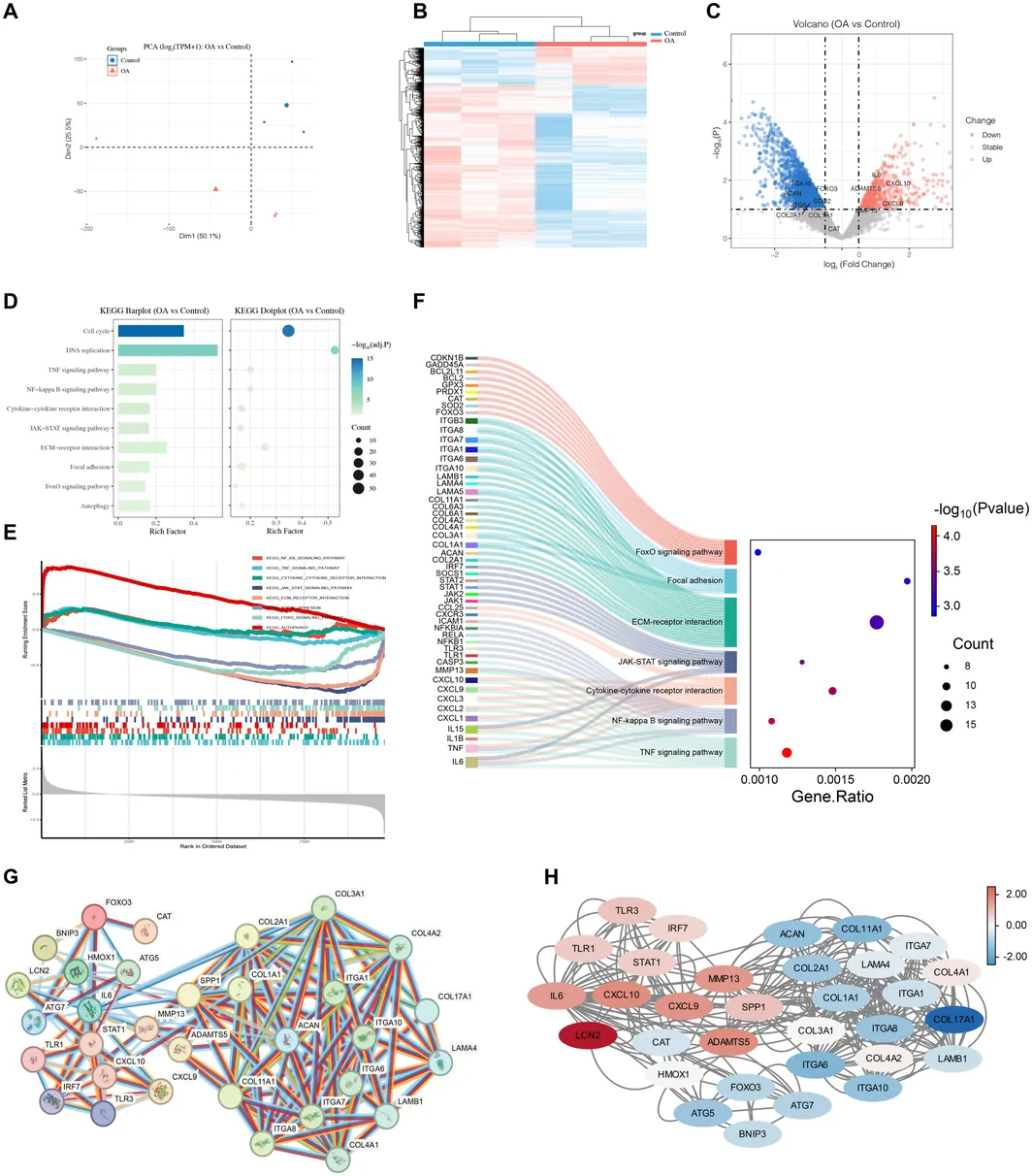
Overview of transcriptomic alterations between OA and Control groups. (A) PCA of RNA‐seq samples. (B) Heatmap of representative gene expression patterns. (C) Volcano plot of differentially expressed genes with selected inflammation‐, ECM‐, integrin‐, and FoxO3‐related genes highlighted. (D) KEGG over‐representation analysis shown as barplot and dotplot. (E) GSEA of KEGG pathways. (F) Sankey and bubble plots linking enriched pathways to associated genes. (G) STRING‐derived PPI network. (H) Cytoscape visualization of the PPI network with incorporated differential expression information.

Principal component analysis showed clear separation between control and OA samples along the first principal component, indicating distinct transcriptional profiles (Figure 1A). Heatmap analysis confirmed tight within‐group clustering and prominent inter‐group differences (Figure 1B). Differential expression analysis identified 578 up‐regulated and 2476 down‐regulated genes. Key OA‐related genes included elevated inflammatory mediators (IL6, CXCL9, CXCL10, STAT1), catabolic enzymes (MMP13, ADAMTS5), and reduced extracellular matrix and integrin genes (ACAN, COL2A1, COL1A1, ITGA6, ITGA8, ITGA10), as well as FOXO3‐associated autophagy/oxidative stress regulators (FOXO3, ATG5, BNIP3, CAT) (Figure 1C). KEGG over‐representation analysis highlighted pathways related to inflammation, cytokine‐receptor interaction, Toll‐like receptor signalling, ECM organization, focal adhesion, and FoxO signalling (Figure 1D). GSEA showed positive enrichment of TNF/NF‐κB/chemokine signalling and apoptosis in OA, while FoxO signalling, autophagy, oxidative stress response, ECM‐receptor interaction and focal adhesion pathways exhibited negative enrichment (Figure 1E). Sankey and bubble plots visualized gene‐pathway associations identified by ORA and GSEA (Figure 1F). STRING analysis identified functional modules related to inflammation/cytokines (IL6, CXCL9, CXCL10, STAT1), catabolism (MMP13, ADAMTS5), ECM/adhesion (ACAN, COL2A1, COL1A1, ITGA6, ITGA8, ITGA10), and FOXO3‐associated autophagy/oxidative stress (FOXO3, ATG5, BNIP3, CAT) (Figure 1G). Cytoscape network integration of log2 fold change profiles further verified the significant upregulation of pro‐inflammatory and catabolic genes, alongside the suppression of genes associated with extracellular matrix formation, integrin signalling, and the FOXO3 regulatory axis (Figure 1H).

### 3.2 Protective Effect of FoxO3 Overexpression on Articular Cartilage in Rats With TMJOA

**FIGURE 2:**
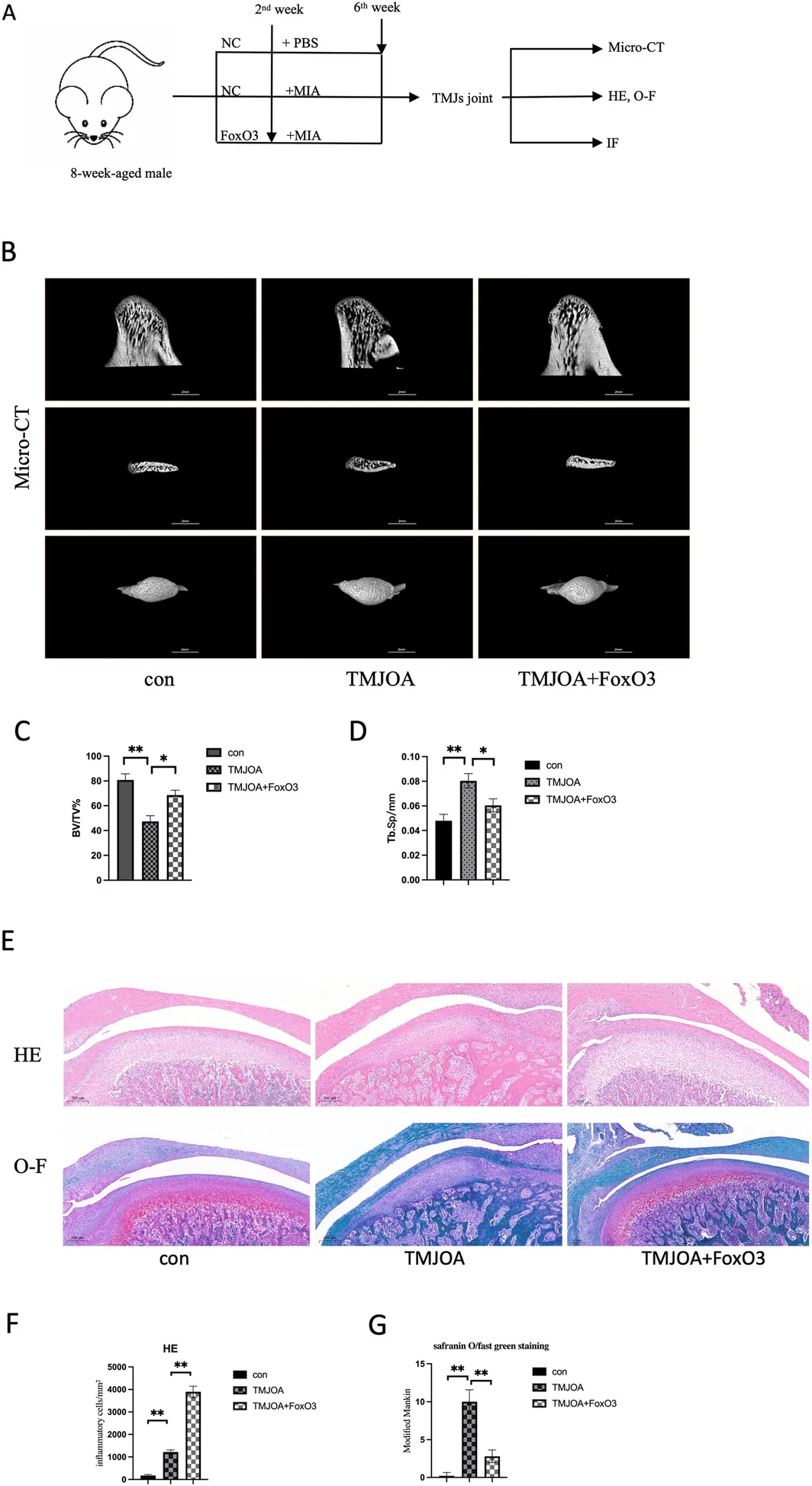
FoxO3 overexpression attenuates the progression of TMJOA phenotypes in rats. (A) Schematic diagram of the animal experimental design. (B) Micro‐CT images of temporomandibular joint specimens from the three groups, showing sagittal views, subchondral bone views, and top views of the TMJ. (C) Quantitative assessment of BV/TV. (D) Quantitative analysis of trabecular separation (Tb.Sp). (E) H&E staining and Safranin O‐fast green staining (O–F) of rat TMJs. (F) Histological scoring of neutrophil infiltration in rat TMJs. (G) OARSI scoring of rat TMJs. (*p < 0.05, **p < 0.01; *n* = 3).

**FIGURE 3:**
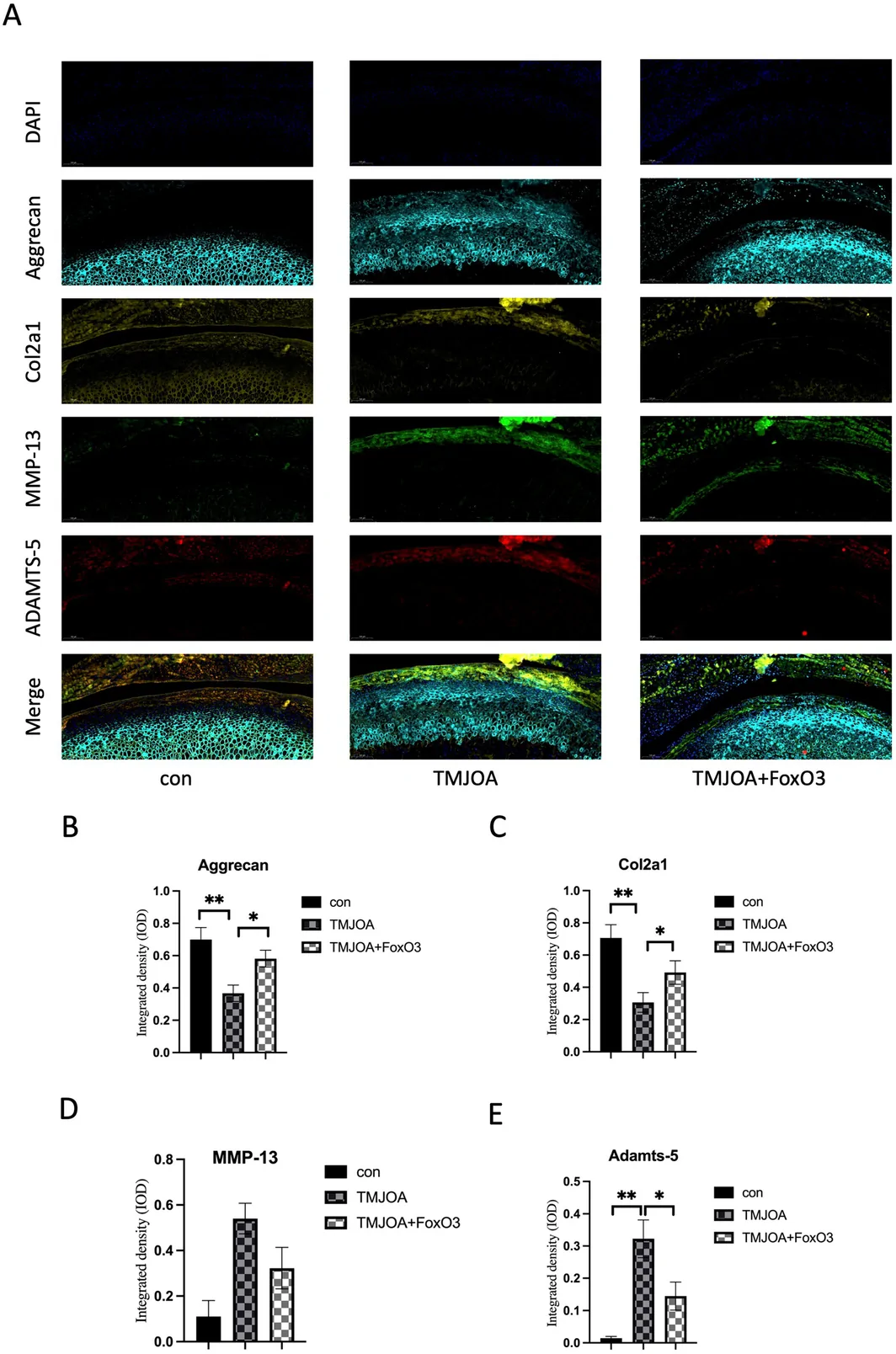
Immunofluorescence staining and quantification of cartilage inflammation‐ and matrix‐related genes in the TMJ of rats from the three groups. (A) Representative immunofluorescence images of Aggrecan, Col2a1, MMP‐13, ADAMTS‐5, and DAPI staining, as well as merged images, in TMJ cartilage from the three groups. (B–E) Quantitative analysis of the fluorescence intensity for Aggrecan (B), Col2a1 (C), MMP‐13 (D), and ADAMTS‐5 (E). (*p < 0.05, **p < 0.01; *n* = 3).

Rats model with TMJ cells FoxO3 overexpression was constructed using AAV5‐CAG‐rFoxO3‐IRES‐EGFP. 2 weeks later, the TMJOA rat model was established by MIA injection, and the rats were divided into 3 groups: control group (Con group), TMJOA group (empty vector + MIA injection), and TMJOA+FoxO3 group (FoxO3 overexpression + MIA injection). After 4 weeks of observation, the temporomandibular joints of rats in the three groups were harvested for phenotypic detection and pathological staining (Figure 2A). Micro‐CT analysis demonstrated that the subchondral bone volume fraction of the temporomandibular joint was markedly increased in the TMJOA+FoxO3 group relative to the untreated TMJOA group, accompanied by a remarkable reduction in trabecular separation (*p* < 0.05) (Figure 2B). Histological staining indicated that the TMJOA+FoxO3 group had a more intact articular cartilage surface, fewer inflammatory cells, higher matrix proteoglycan content, and a significantly lower cartilage damage score compared with the TMJOA group (*p* < 0.05) (Figure 2E). Further immunofluorescence staining of rat TMJ cartilage revealed that the expression levels of MMP‐13 and ADAMTS‐5 were significantly downregulated in the TMJOA+FoxO3 group compared with the TMJOA group. In contrast, the cartilage matrix markers Col2a1 and Aggrecan were markedly upregulated following FoxO3 overexpression (*p* < 0.05) (Figure 3A). Consistent with the above findings, FoxO3 intervention effectively attenuated chondrocyte inflammatory catabolism and restored extracellular matrix synthesis in TMJOA lesions.

### 3.3 Protective Role of FoxO3 Gene and Protein in Chondrocytes

**FIGURE 4:**
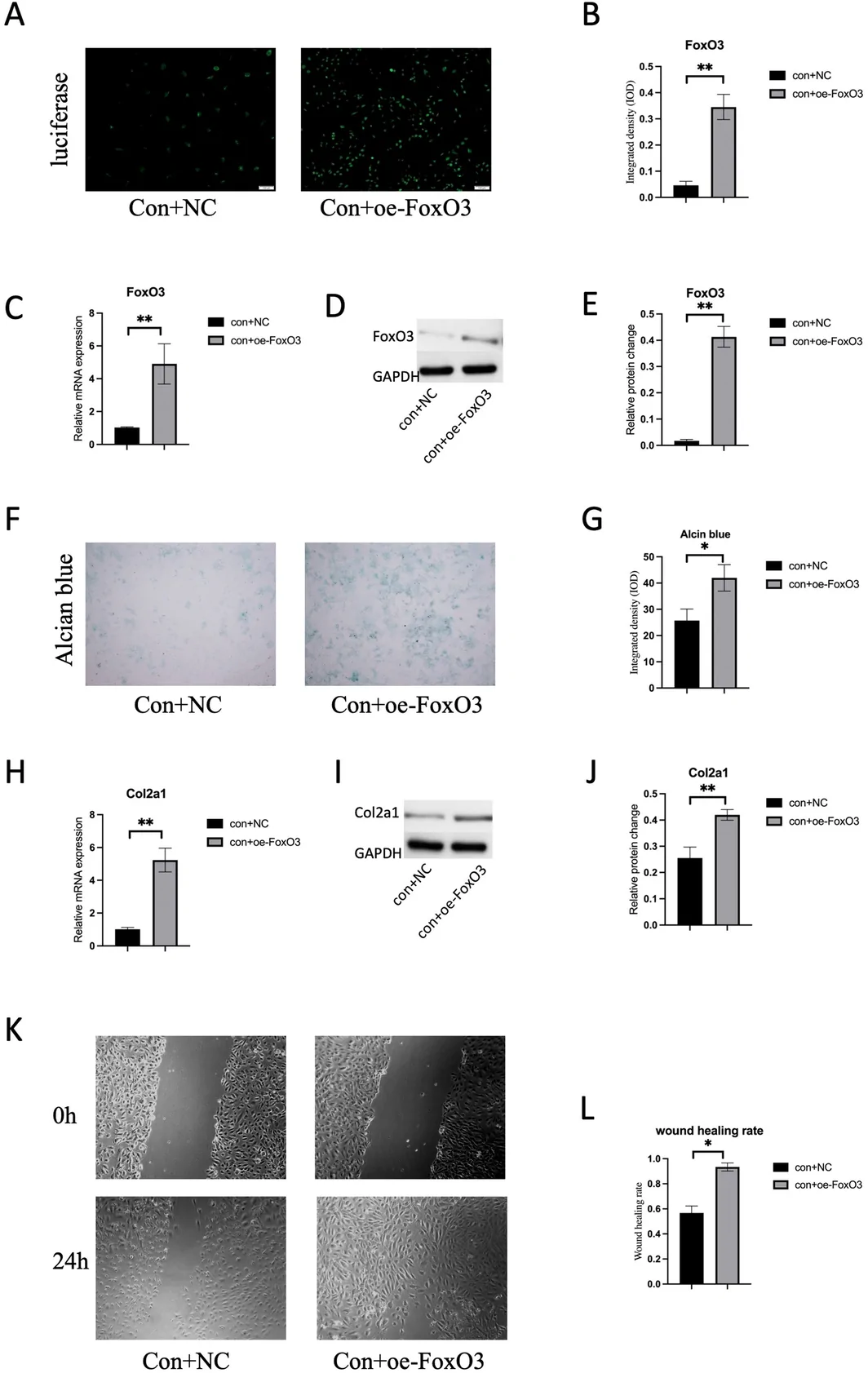
FoxO3 overexpression promotes chondrocyte matrix synthesis and secretion, Col2a1 expression, and cell proliferation. (A) Fluorescence images showing normal expression of the FoxO3‐overexpression plasmid in chondrocytes. (B) Quantitative analysis of fluorescence intensity. (C) QRT‐PCR confirming successful overexpression of FoxO3 in chondrocytes. (D) Western blot showing increased FoxO3 protein expression. (E) Quantification of western blot results. (F) Alcian blue staining indicating enhanced matrix synthesis and secretion in FoxO3‐overexpressing chondrocytes. (G) Quantification of Alcian blue staining. (H) QRT‐PCR showing upregulated Col2a1 expression in FoxO3‐overexpressing chondrocytes. (I) Western blot confirming increased Col2a1 protein levels. J: Quantification of western blot results. (K) 24‐h scratch assay demonstrating enhanced chondrocyte proliferation upon FoxO3 overexpression. (L) Quantification of the scratch assay. (*p < 0.05, **p < 0.01; *N* = 3.)

To clarify the specific role of the FoxO3 gene in chondrocytes, FoxO3 overexpression plasmids were transfected into primary rat chondrocytes. Fluorescence staining confirmed successful plasmid expression (Figure 4A,B), and consistent alterations in both mRNA and protein expression of FoxO3 were observed between groups, with statistically significant differences (*p* < 0.05) (Figure 4C–E). Alcian blue staining was performed to detect acidic glycosaminoglycan (GAG) deposition in chondrocytes. The staining results demonstrated that FoxO3 overexpression prominently enhanced the matrix synthetic ability of chondrocytes (Figure 4F,G). Meanwhile, immunofluorescence detection further confirmed that FoxO3 modulated the differential expression of Col2a1 protein (Figure 4H–J). A 24‐h cell scratch assay demonstrated that the FoxO3 gene could promote chondrocyte proliferation (*p* < 0.05) (Figure 4K,L).

### 3.4 Regulatory Effect of the lncRNA OIP5‐AS1/Rno‐miRNA‐223‐3p/FoxO3 Axis on Chondrocytes

**FIGURE 5:**
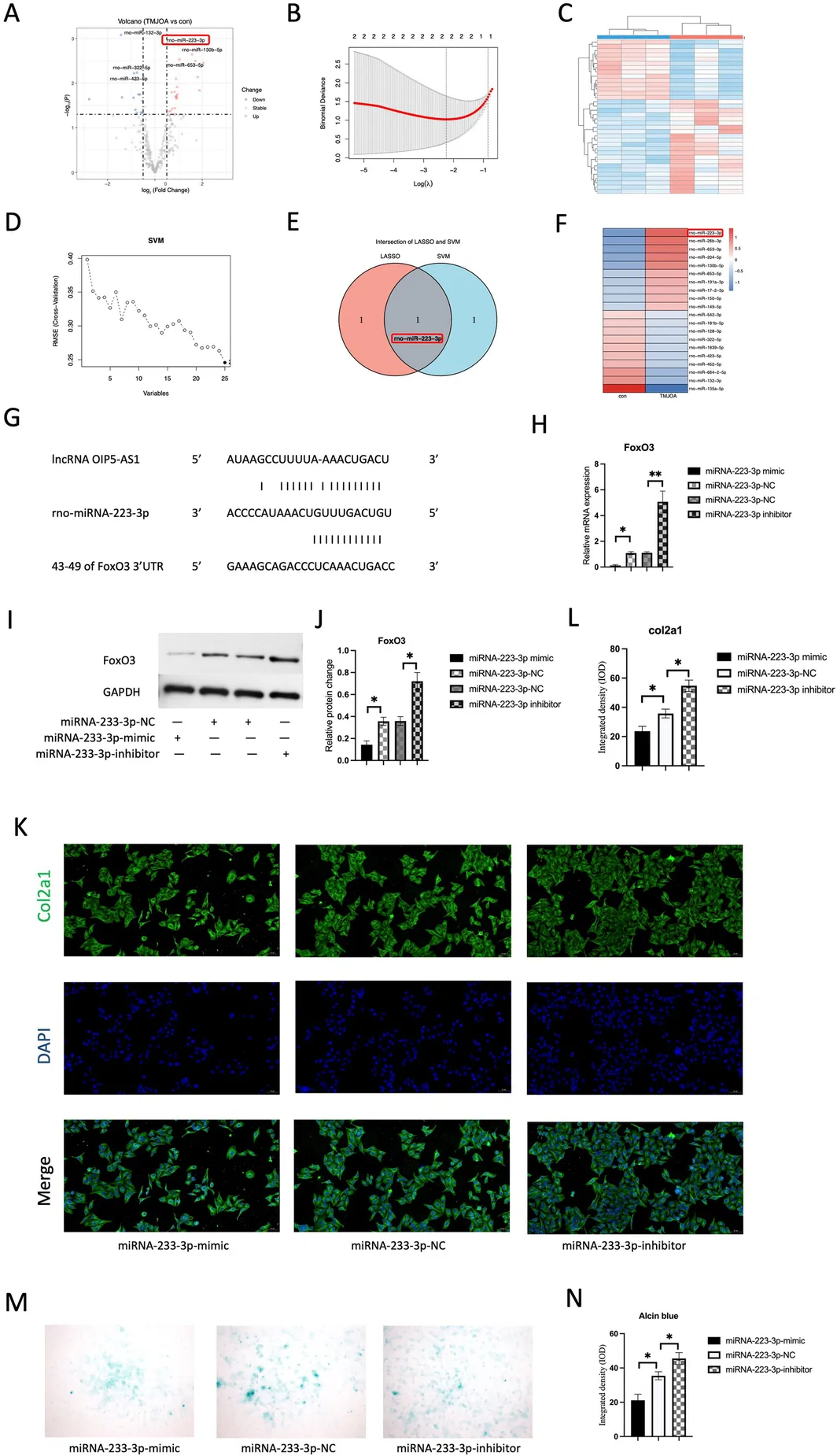
Identification of key miRNAs rno‐miRNA‐223‐3p associated with TMJOA. (A) Volcano plot of differentially expressed miRNAs between TMJOA and control groups, with representative miRNAs highlighted; (B) Heatmap of differentially expressed miRNA expression profiles across TMJOA and control samples; (C) LASSO regression analysis for feature selection of candidate miRNAs; (D) SVM‐based feature selection showing the relationship between variable number and cross‐validation error; (E) Venn diagram showing the intersection of miRNAs identified by LASSO and SVM; (F) Heatmap based on log_2_ fold changes illustrating expression trends of representative differentially expressed miRNAs between TMJOA and control groups. (G) Based on extensive historical literature data, rno‐miRNA‐223‐3p is predicted to competitively bind to lncRNA OIP5‐AS1 and the 3'UTR of FoxO3 through complementary binding sites. (H) Transfection with rno‐miRNA‐223‐3p inhibitor or mimic significantly regulated FoxO3 gene expression and protein translation (I, J). (K) Specific fluorescence staining showed that rno‐miRNA‐223‐3p inhibitor and mimic further affected Col2a1 protein expression in chondrocytes, with L showing the quantitative analysis of Col2a1 fluorescence intensity. (M) Alcian blue staining indicated that rno‐miRNA‐223‐3p inhibitor and mimic further modulated chondrocyte matrix formation and secretion, with N showing the quantitative analysis of Alcian blue staining. (*p < 0.05, **p < 0.01; *N* = 3.)

Small RNA sequencing showed distinct miRNA expression profiles between TMJOA and control groups. Differential analysis identified 34 significantly dysregulated miRNAs (22 up‐regulated, 12 down‐regulated) in TMJOA samples (Figure 5A), and heatmap confirmed clear inter‐group separation as well as consistent miRNA expression differences across biological replicates (Figure 5B). To screen miRNAs with strong discriminatory potential, differentially expressed miRNAs underwent machine learning feature selection: LASSO regression selected two candidates (rno‐miR‐223‐3p, rno‐miR‐653‐5p) (Figure 5C), while a linear SVM model ranked rno‐miR‐223‐3p and rno‐miR‐322‐5p as top features (Figure 5D). Intersection analysis identified rno‐miR‐223‐3p as the only miRNA consistently selected by both methods, thus defined as the key candidate (Figure 5E). A log_2_ fold change‐based heatmap illustrated expression trends of representative differentially expressed miRNAs (Figure 5F), showing distinct up/down‐regulated clusters. Notably, rno‐miR‐223‐3p was significantly up‐regulated in TMJOA samples, while rno‐miR‐653‐5p and rno‐miR‐322‐5p exhibited opposite trends.

Website (https://www.targetscan.org/vert_80/) prediction suggested that rno‐miRNA‐223‐3p could bind to both FoxO3 mRNA and lncRNA OIP5‐AS1. Accumulating evidence has demonstrated that lncRNA OIP5‐AS1 contains binding sites for miRNA‐223‐3p, thereby inhibiting the downstream effects of miRNA‐223‐3p. To clarify the specific downstream functions of rno‐miRNA‐223‐3p, chondrocytes were transfected with rno‐miRNA‐223‐3p mimic or rno‐miRNA‐223‐3p inhibitor. The results showed that the expression of downstream FoxO3 changed accordingly, presenting a negative regulatory relationship (Figure 5H–J). Specifically, rno‐miRNA‐223‐3p could bind to FoxO3 mRNA, inhibit its transcriptional expression, and directly affect the expression of Col2a1 in chondrocytes (Figure 5L,K) and the synthesis of acidic GAGs (Figure 5M,N). These findings indicate that rno‐miRNA‐223‐3p has physiological significance at the transcriptional level (*p* < 0.05).

## 4 Discussion

Temporomandibular joint osteoarthritis (TMJOA) is a prevalent chronic degenerative joint disorder characterized by progressive and irreversible articular cartilage destruction. Its complex pathological cascade encompasses multiple detrimental cellular events, including suppressed chondrocyte proliferation, aberrant chondrocyte apoptosis, disrupted homeostasis of extracellular matrix anabolism and catabolism, and persistent synovial inflammation. To date, the exact molecular mechanisms underlying TMJOA pathogenesis remain incompletely clarified, and definitive curative clinical therapies for TMJOA are still lacking. In this context, in‐depth exploration of the key molecular regulators governing chondrocyte functional homeostasis is essential to uncover novel therapeutic targets and establish theoretical foundations for targeted TMJOA intervention. Long non‐coding RNAs (lncRNAs) are a subclass of non‐protein‐coding RNAs longer than 200 nucleotides. Functionally, lncRNAs act as competing endogenous RNAs (ceRNAs) to modulate the expression of microRNAs (miRNAs) and their downstream target genes, thereby exerting pivotal regulatory effects on the progression of osteoarthritic cartilage degeneration [12, 13].

This study focused on the lncRNA OIP5‐AS1/rno‐miRNA‐223‐3p/FoxO3 axis, exploring its regulatory effects on chondrocyte proliferation, apoptosis, and matrix metabolism, aiming to reveal a novel molecular mechanism underlying TMJOA pathogenesis.

Long non‐coding RNA OIP5‐AS1 (OIP5 antisense RNA 1), located at the human chromosome 15q21.1 locus, was initially characterized in oncological studies. Dysregulated expression of OIP5‐AS1 is tightly correlated with malignant phenotypes, including tumour proliferation, apoptosis, invasion, and metastasis [14]. Notably, emerging evidence has expanded its functional spectrum, demonstrating that OIP5‐AS1 also serves as a vital regulatory molecule in the occurrence and progression of bone and joint degenerative diseases. Existing studies have confirmed that OIP5‐AS1 exerts chondroprotective effects by modulating chondrocyte viability, inflammatory response, extracellular matrix metabolism, and osteogenic differentiation, suggesting its promising therapeutic potential in osteoarthritis‐related pathological processes [15]. Accumulating clinical and in vitro evidence has verified that OIP5‐AS1 is markedly downregulated in the cartilage tissues of OA patients, as well as in chondrocyte models induced by IL‐1β. Moreover, the decreased expression of OIP5‐AS1 presents a dose‐ and time‐dependent pattern, with more significant downregulation observed following increased IL‐1β concentrations and prolonged stimulation duration. Zhi et al. found in lines that after stimulated with serial concentrations of IL‐1β (1, 5, and 10 ng/mL), the mRNA expression of OIP5‐AS1 decreased in a gradient manner, accompanied by reduced chondrocyte proliferation capacity, increased apoptosis rate, and elevated inflammatory factor secretion, suggesting that IL‐1β may initiate the cartilage degeneration process by inhibiting OIP5‐AS1 expression [16]. Consistent with these findings, independent research has also validated suppressed OIP5‐AS1 expression in IL‐1β‐treated primary chondrocytes and CHON‐001 cell lines under inflammatory stimulation; OIP5‐AS1 knockdown mimics IL‐1β‐induced chondrocyte injury and pro‐catabolic effects, whereas OIP5‐AS1 overexpression efficiently rescues the IL‐1β‐mediated detrimental alterations in chondrocytes. These findings solidly confirm the negative regulatory correlation between IL‐1β and OIP5‐AS1 in chondrocyte inflammatory injury [15]. Although the precise molecular mechanism by which IL‐1β represses OIP5‐AS1 expression remains poorly defined, this regulatory pattern is presumed to be closely associated with IL‐1β‐triggered activation of the NF‐κB and PI3K/Akt signalling cascades. Accumulating evidence has validated that IL‐1β modulates the transcription of downstream lncRNAs predominantly via NF‐κB signalling activation. In addition, bioinformatic prediction has identified potential NF‐κB binding motifs within the promoter region of OIP5‐AS1. Collectively, these findings imply that IL‐1β may transcriptionally suppress OIP5‐AS1 activity through direct modulation of the NF‐κB pathway in chondrocytes [17]. This speculation awaits further verification by subsequent promoter activity analysis experiments.

As core regulatory mediators of the ceRNA network, microRNAs (miRNAs) modulate target gene expression at the post‐transcriptional level via sequence‐specific binding, thereby participating in the maintenance of chondrocyte functional homeostasis and regulating osteoarthritis progression [18]. In the MIA‐induced rat model of TMJOA, rno‐miRNA‐223‐3p is significantly upregulated, and literature has indicated that lncRNA OIP5‐AS1 shares competitive binding sites with miRNA‐223‐3p [17]. As a well‐characterized regulatory microRNA, rno‐miR‐223‐3p participates extensively in the modulation of inflammatory responses, cell proliferation, and apoptotic processes. Emerging evidence has demonstrated markedly elevated miR‐223‐3p expression in OA cartilage tissues. Functionally, miR‐223‐3p drives chondrocyte catabolic dysfunction by suppressing cell proliferation, facilitating chondrocyte apoptosis, and exacerbating cartilage degenerative lesions, predominantly through targeted inhibition of FoxO1 signalling [19]. Furthermore, these discrepant findings indicate that the biological function of miR‐223‐3p in OA progression may be tissue‐specific and context‐dependent, varying according to downstream target profiles [20, 21]. In the present study, bioinformatic prediction combined with dual‐luciferase reporter assays validated that rno‐miR‐223‐3p directly targets and binds to the 3′ untranslated region (3′UTR) of FoxO3 mRNA. Functional validation experiments demonstrated that inhibition of rno‐miR‐223‐3p significantly elevates FoxO3 expression in chondrocytes and modulates the transcription of its downstream target genes. Consistently, functional phenotypic experiments revealed that rno‐miR‐223‐3p overexpression represses chondrocyte proliferation and accelerates extracellular matrix catabolism. Collectively, these data clarify the molecular mechanism by which rno‐miR‐223‐3p mediates chondrocyte functional homeostasis via targeted regulation of FoxO3 signalling during TMJOA progression.

Therefore, after the upregulation of miRNA‐223‐3p, it is highly likely to bind to lncRNA OIP5‐AS1, leading to a decrease in free lncRNA OIP5‐AS1, thereby activating inflammatory pathways and inducing an increase in inflammatory markers. This expands the regulatory mechanism of lncRNA OIP5‐AS1. Existing data indicate that rno‐miR‐223‐3p can activate inflammatory responses and inhibit cartilage matrix production by suppressing FoxO3 expression [22]. Thus, the reduction of free lncRNA OIP5‐AS1 results in the upregulation of rno‐miR‐223‐3p, which competitively inhibits FoxO3 expression, complementing the regulatory mechanism of FoxO3.

As an important member of the FoxO family, Forkhead Box O3 (FoxO3) is an evolutionarily conserved transcription factor. By modulating the transcription of downstream target genes, FoxO3 participates in the regulation of diverse physiological and pathological processes, including cell cycle progression, apoptosis, oxidative stress response, and energy metabolism [5]. In cartilage tissue, FoxO3 has been confirmed as a key regulatory factor maintaining chondrocyte functional homeostasis: FoxO3 can enhance the antioxidant stress capacity of chondrocytes and reduce cell apoptosis by activating downstream target genes (such as SOD2 and CAT) [23]. Meanwhile, FoxO3 exerts chondroprotective effects by facilitating the synthesis of cartilage matrix biomarkers Col2a1 and Aggrecan, suppressing the expression of MMP family catabolic genes, and thereby preserving the structural integrity of the cartilage extracellular matrix [5]. During the pathological progression of OA, the expression and activity of FoxO3 are significantly reduced, leading to weakened antioxidant capacity and increased apoptosis of chondrocytes, ultimately triggering cartilage degeneration. Consistent with prior reports [4, 24], loss‐of‐function of FoxO3 in joint tissues accelerates osteoarthritic deterioration, showing aggravated cartilage erosion, increased chondrocyte apoptosis, suppressed matrix synthesis and enhanced catabolic activity. Our in vitro gain‐of‐function data complement these findings and verify the therapeutic potential of FoxO3 upregulation for TMJOA. In this study, integrated bioinformatic prediction and experimental validation confirmed that rno‐miR‐223‐3p directly binds to the 3′UTR of FoxO3. Furthermore, rno‐miR‐223‐3p overexpression markedly reduces both mRNA and protein levels of FoxO3, demonstrating that FoxO3 serves as a direct downstream target of rno‐miR‐223‐3p in chondrocytes. These results confirm the critical regulatory role of the lncRNA OIP5‐AS1/rno‐miRNA‐223‐3p/FoxO3 axis in TMJOA. Consistent with existing research conclusions [4], FoxO3 exerts a chondroprotective effect in osteoarthritis, protecting chondrocytes through mechanisms such as antioxidant stress, regulation of autophagy/mitophagy, inhibition of inflammation and ferroptosis, maintenance of extracellular matrix (ECM) homeostasis, and anti‐apoptosis, thereby playing a central role in pathological processes such as osteoarthritis [5]. This study complements the FoxO3‐mediated chondroprotective pathway, providing a potential theoretical basis for the targeted treatment of TMJOA.

Despite the above findings, several limitations of the present study should be acknowledged. First, the mechanism exploration was mainly based on in vitro chondrocyte models, lacking verification in vivo animal experiments. Of note, transfection of rno‐miR‐223‐3p mimic leads to endogenous FoxO3 repression, which functionally mimics FoxO3 knockdown in primary chondrocytes. The resultant impairment of matrix synthesis and Col2a1 down‐regulation indirectly supports the essential role of FoxO3 in maintaining chondrocyte homeostasis. In the future, animal models with OIP5‐AS1 silencing or overexpression should be constructed, combined with the MIA‐induced TMJOA model, to further verify the in vivo regulatory role and therapeutic potential of this axis. Second, this study only focused on the OIP5‐AS1/rno‐miR‐223‐3p/FoxO3 axis and did not explore its crosstalk with other signalling pathways (such as PI3K/Akt and NF‐κB pathways). Since the activity of FoxO3 is often regulated by phosphorylation modification of the PI3K/Akt pathway, in‐depth research on the synergistic regulatory mechanisms between various pathways is needed in the future. Finally, this study did not detect the expression correlation of OIP5‐AS1, rno‐miR‐223‐3p, and FoxO3 in clinical samples. In the future, the sample size of clinical cases should be expanded to verify the expression pattern of this axis in clinical TMJOA patients, providing a basis for its clinical application. Apart from articular chondrocytes, rno‐miR‐223‐3p and FoxO3 may exert regulatory effects on multiple cell types within TMJ microenvironment. According to previous osteoarthritis studies [25, 26], synovial macrophages, synovial fibroblasts, subchondral osteoblasts and osteoclasts jointly drive synovitis, subchondral bone remodelling and whole‐joint deterioration during OA progression. Existing literature reported that miR‐223‐3p is a well‐known regulator of macrophage polarization: elevated miR‐223‐3p can promote pro‐inflammatory M1 macrophage phenotype and facilitate synovial inflammation. FoxO3 participates in inhibiting macrophage inflammatory activation, regulates osteoblast differentiation and restrains osteoclast over‐activation. Therefore, the lncRNA‐OIP5‐AS1/rno‐miR‐223‐3p/FoxO3 axis may potentially regulate synovial inflammation and subchondral bone remodelling beyond chondrocyte apoptosis. However, the present work only characterized its function in chondrocytes; further cellular and animal experiments are required to clarify its roles in macrophages, synovial fibroblasts and bone cells in TMJOA.

In conclusion, rno‐miR‐223‐3p downregulates FoxO3 expression by competitively binding to FoxO3 mRNA, promotes chondrocyte matrix degradation, inhibits cell proliferation, and thereby participates in the cartilage degeneration process of TMJOA. Collectively, the newly identified lncRNA OIP5‐AS1/rno‐miR‐223‐3p/FoxO3 regulatory axis holds great promise as a novel molecular biomarker for diagnosis and an effective therapeutic target for TMJOA, offering an innovative theoretical basis and actionable strategy for precise clinical intervention against TMJOA.

## Author Contributions

**Xuesong Xu:** methodology, validation, visualization, investigation, writing – original draft, formal analysis, software. **Shichong Qiao:** conceptualization, writing – review and editing, project administration, supervision, data curation.

## Funding

This work was supported by Horizontal project (SC20231015‐9H).

## Ethics Statement

This study adhered strictly to ethical guidelines and regulations concerning animal experiments. All procedures were approved by the Institutional Animal Care and Use Committee (IACUC) (Ethical Approval by the Institutional Animal Care and Use Committee of Huadong Hospital). All animals were housed and cared for in a humane environment, with efforts made to minimize suffering. At the end of the experiments, all rats were humanely euthanized under ether anaesthesia.

## Conflicts of Interest

The authors declare no conflicts of interest.

## Supporting information


**Table S1:** AAV5‐CAG‐rFoxO3‐IRES‐EGFP.
**Table S2:** Summary of primers for rat RT‐qPCR.

## Data Availability

The data that support the findings of this study are available from the corresponding author upon reasonable request.
