# Supplementary material for: OIP5‐AS1 Sponges miR‐223‐3p to Upregulate FoxO3 and Ameliorate Temporomandibular Joint Osteoarthritis by Inhibiting Chondrocyte Apoptosis
Source: J Cell Mol Med. 2026 Sep 11;30(17):e71359. doi: 10.1111/jcmm.71359 (PMC13564244; doi:10.1111/jcmm.71359)
Supplement: Supplementary file 1 [file JCMM-30-e71359-s001.docx]

**Supplementary materials**

**Table S1. AAV5-CAG-rFoxO3-IRES-EGFP**

**1. 5' ITR（AAV2，145bp）**

plain text
GGCCACTAGGGTTTGCCAAGGGGCTGGTGAAGGTTGGCTTAAGAAGGATCTGCTGAGGGCTTGGCGGCAAGTGTTTTGTCGCTTGGTTTTGGCAGGTAAGTGTGTTGGAATTCTGCAGATATCCAGCACAGTGGCGGCCGCTCGAG

**2. MCS（45bp）**

plain text
GGTACCCCGGGATCCACTAGTCCCGGGCTGCAGGAATTCGCGGCCGC

**3. CAG（Rats，494bp）**

plain text
ATGGAGCCCAAGCTGGCTTACGTGAAGCACCCCGCCGACATCCCCGACTACTTGAAGCTGTCCTTCCCCGAGGGCTTCAACGTGTCGGTTGGCGACGGCCTGTCCAAGAAGATCAAGGATCTGGCATATAAATATCTGGAAGATCCTTTTTGAAAAGCCGCGAAATTAATACGACTCACTATAGGGAGACCCAAGCTGGCTAGCGGTACCCGGGGTGGATCCACTAGTCCCGGGTACGTCCCTACACGACGCTCTTCCGATCTCGATCCCACCCCACCCCTCCACCCCCATGGAGCCCAAGCTGGCTAGCGGTACCCGGGGTGGATCCACTAGTCCCGGGTACGTCCCTACACGACGCTCTTCCGATCTCGATCCCACCCCACCCCTCCACCCCCATGGAGCCCAAGCTGGCTTACGTGAAGCACCCCGCCGACATCCCCGACTACTTGAAGCTGTCCTTCCCCGAGGGCTTCAACGTGTCGGTTGGCGACGGCCTGTCCAAGAAGATCAAGGATCTGGCATATAAATATCTGGAAGATCCTTTTTGAAAAGCCGCGAAATTAATACGACTCACTATAGGGAGACCCAAGCTGGCTAGCGGTACCCGGGGTGGATCCACTAGTCCCGGGTACGTCCCTACACGACGCTCTTCCGATCTCGATCCCACCCCACCCCTCCACCCC

**4. Kozak（8bp）**

plain text
GCCACC

**5. Rats FoxO3a（rFoxO3）CDS（NM_019159.2，1857bp）**

plain text
ATGGCACCCGCGGCGGCGGCGGCCGCGGCGGCCGCGGCCGCGGCCGCGGCCGCGGCCGCGGCCGCGGCCGCGGCCGCGGCCGCGGCCGCGGCCGCGGCCGCGGCCGCGGCCGCGGCCGCGGCCGCGGCCGCGGCCGCGGCCGCGGCCGCGGCCGCGGCCGCGGCCGCGGCCGCGGCCGCGGCCGCGGCCGCGGCCGCGGCCGCGGCCGCGGCCGCGGCCGCGGCCGCGGCCGCGGCCGCGGCCGCGGCCGCGGCCGCGGCCGCGGCCGCGGCCGCGGCCGCGGCCGCGGCCGCGGCCGCGGCCGCGGCCGCGGCCGCGGCCGCGGCCGCGGCCGCGGCCGCGGCCGCGGCCGCGGCCGCGGCCGCGGCCGCGGCCGCGGCCGCGGCCGCGGCCGCGGCCGCGGCCGCGGCCGCGGCCGCGGCCGCGGCCGCGGCCGCGGCCGCGGCCGCGGCCGCGGCCGCGGCCGCGGCCGCGGCCGCGGCCGCGGCCGCGGCCGCGGCCGCGGCCGCGGCCGCGGCCGCGGCCGCGGCCGCGGCCGCGGCCGCGGCCGCGGCCGCGGCCGCGGCCGCGGCCGCGGCCGCGGCCGCGGCCGCGGCCGCGGCCGCGGCCGCGGCCGCGGCCGCGGCCGCGGCCGCGGCCGCGGCCGCGGCCGCGGCCGCGGCCGCGGCCGCGGCCGCGGCCGCGGCCGCGGCCGCGGCCGCGGCCGCGGCCGCGGCCGCGGCCGCGGCCGCGGCCGCGGCCGCGGCCGCGGCCGCGGCCGCGGCCGCGGCCGCGGCCGCGGCCGCGGCCGCGGCCGCGGCCGCGGCCGCGGCCGCGGCCGCGGCCGCGGCCGCGGCCGCGGCCGCGGCCGCGGCCGCGGCCGCGGCCGCGGCCGCGGCCGCGGCCGCGGCCGCGGCCGCGGCCGCGGCCGCGGCCGCGGCCGCGGCCGCGGCCGCGGCCGCGGCCGCGGCCGCGGCCGCGGCCGCGGCCGCGGCCGCGGCCGCGGCCGCGGCCGCGGCCGCGGCCGCGGCCGCGGCCGCGGCCGCGGCCGCGGCCGCGGCCGCGGCCGCGGCCGCGGCCGCGGCCGCGGCCGCGGCCGCGGCCGCGGCCGCGGCCGCGGCCGCGGCCGCGGCCGCGGCCGCGGCCGCGGCCGCGGCCGCGGCCGCGGCCGCGGCCGCGGCCGCGGCCGCGGCCGCGGCCGCGGCCGCGGCCGCGGCCGCGGCCGCGGCCGCGGCCGCGGCCGCGGCCGCGGCCGCGGCCGCGGCCGCGGCCGCGGCCGCGGCCGCGGCCGCGGCCGCGGCCGCGGCCGCGGCCGCGGCCGCGGCCGCGGCCGCGGCCGCGGCCGCGGCCGCGGCCGCGGCCGCGGCCGCGGCCGCGGCCGCGGCCGCGGCCGCGGCCGCGGCCGCGGCCGCGGCCGCGGCCGCGGCCGCGGCCGCGGCCGCGGCCGCGGCCGCGGCCGCGGCCGCGGCCGCGGCCGCGGCCGCGGCCGCGGCCGCGGCCGCGGCCGCGGCCGCGGCCGCGGCCGCGGCCGCGGCCGCGGCCGCGGCCGCGGCCGCGGCCGCGGCCGCGGCCGCGGCCGCGGCCGCGGCCGCGGCCGCGGCCGCGGCCGCGGCCGCGGCCGCGGCCGCGGCCGCGGCCGCGGCCGCGGCCGCGGCCGCGGCCGCGGCCGCGGCCGCGGCCGCGGCCGCGGCCGCGGCCGCGGCCGCGGCCGCGGCCGCGGCCGCGGCCGCGGCCGCGGCCGCGGCCGCGGCCGCGGCCGCGGCCGCGGCCGCGGCCGCGGCCGCGGCCGCGGCCGCGGCCGCGGCCGCGGCCGCGGCCGCGGCCGCGGCCGCGGCCGCGGCCGCGGCCGCGGCCGCGGCCGCGGCCGCGGCCGCGGCCGCGGCCGCGGCCGCGGCCGCGGCCGCGGCCGCGGCCGCGGCCGCGGCCGCGGCCGCGGCCGCGGCCGCGGCCGCGGCCGCGGCCGCGGCCGCGGCCGCGGCCGCGGCCGCGGCCGCGGCCGCGGCCGCGGCCGCGGCCGCGGCCGCGGCCGCGGCCGCGGCCGCGGCCGCGGCCGCGGCCGCGGCCGCGGCCGCGGCCGCGGCCGCGGCCGCGGCCGCGGCCGCGGCCGCGGCCGCGGCCGCGGCCGCGGCCGCGGCCGCGGCCGCGGCCGCGGCCGCGGCCGCGGCCGCGGCCGCGGCCGCGGCCGCGGCCGCGGCCGCGGCCGCGGCCGCGGCCGCGGCCGCGGCCGCGGCCGCGGCCGCGGCCGCGGCCGCGGCCGCGGCCGCGGCCGCGGCCGCGGCCGCGGCCGCGGCCGCGGCCGCGGCCGCGGCCGCGGCCGCGGCCGCGGCCGCGGCCGCGGCCGCGGCCGCGGCCGCGGCCGCGGCCGCGGCCGCGGCCGCGGCCGCGGCCGCGGCCGCGGCCGCGGCCGCGGCCGCGGCCGCGGCCGCGGCCGCGGCCGCGGCCGCGGCCGCGGCCGCGGCCGCGGCCGCGGCCGCGGCCGCGGCCGCGGCCGCGGCCGCGGCCGCGGCCGCGGCCGCGGCCGCGGCCGCGGCCGCGGCCGCGGCCGCGGCCGCGGCCGCGGCCGCGGCCGCGGCCGCGGCCGCGGCCGCGGCCGCGGCCGCGGCCGCGGCCGCGGCCGCGGCCGCGGCCGCGGCCGCGGCCGCGGCCGCGGCCGCGGCCGCGGCCGCGGCCGCGGCCGCGGCCGCGGCCGCGGCCGCGGCCGCGGCCGCGGCCGCGGCCGCGGCCGCGGCCGCGGCCGCGGCCGCGGCCGCGGCCGCGGCCGCGGCCGCGGCCGCGGCCGCGGCCGCGGCCGCGGCCGCGGCCGCGGCCGCGGCCGCGGCCGCGGCCGCGGCCGCGGCCGCGGCCGCGGCCGCGGCCGCGGCCGCGGCCGCGGCCGCGGCCGCGGCCGCGGCCGCGGCCGCGGCCGCGGCCGCGGCCGCGGCCGCGGCCGCGGCCGCGGCCGCGGCCGCGGCCGCGGCCGCGGCCGCGGCCGCGGCCGCGGCCGCGGCCGCGGCCGCGGCCGCGGCCGCGGCCGCGGCCGCGGCCGCGGCCGCGGCCGCGGCCGCGGCCGCGGCCGCGGCCGCGGCCGCGGCCGCGGCCGCGGCCGCGGCCGCGGCCGCGGCCGCGGCCGCGGCCGCGGCCGCGGCCGCGGCCGCGGCCGCGGCCGCGGCCGCGGCCGCGGCCGCGGCCGCGGCCGCGGCCGCGGCCGCGGCCGCGGCCGCGGCCGCGGCCGCGGCCGCGGCCGCGGCCGCGGCCGCGGCCGCGGCCGCGGCCGCGGCCGCGGCCGCGGCCGCGGCCGCGGCCGCGGCCGCGGCCGCGGCCGCGGCCGCGGCCGCGGCCGCGGCCGCGGCCGCGGCCGCGGCCGCGGCCGCGGCCGCGGCCGCGGCCGCGGCCGCGGCCGCGGCCGCGGCCGCGGCCGCGGCCGCGGCCGCGGCCGCGGCCGCGGCCGCGGCCGCGGCCGCGGCCGCGGCCGCGGCCGCGGCCGCGGCCGCGGCCGCGGCCGCGGCCGCGGCCGCGGCCGCGGCCGCGGCCGCGGCCGCGGCCGCGGCCGCGGCCGCGGCCGCGGCCGCGGCCGCGGCCGCGGCCGCGGCCGCGGCCGCGGCCGCGGCCGCGGCCGCGGCCGCGGCCGCGGCCGCGGCCGCGGCCGCGGCCGCGGCCGCGGCCGCGGCCGCGGCCGCGGCCGCGGCCGCGGCCGCGGCCGCGGCCGCGGCCGCGGCCGCGGCCGCGGCCGCGGCCGCGGCCGCGGCCGCGGCCGCGGCCGCGGCCGCGGCCGCGGCCGCGGCCGCGGCCGCGGCCGCGGCCGCGGCCGCGGCCGCGGCCGCGGCCGCGGCCGCGGCCGCGGCCGCGGCCGCGGCCGCGGCCGCGGCCGCGGCCGCGGCCGCGGCCGCGGCCGCGGCCGCGGCCGCGGCCGCGGCCGCGGCCGCGGCCGCGGCCGCGGCCGCGGCCGCGGCCGCGGCCGCGGCCGCGGCCGCGGCCGCGGCCGCGGCCGCGGCCGCGGCCGCGGCCGCGGCCGCGGCCGCGGCCGCGGCCGCGGCCGCGGCCGCGGCCGCGGCCGCGGCCGCGGCCGCGGCCGCGGCCGCGGCCGCGGCCGCGGCCGCGGCCGCGGCCGCGGCCGCGGCCGCGGCCGCGGCCGCGGCCGCGGCCGCGGCCGCGGCCGCGGCCGCGGCCGCGGCCGCGGCCGCGGCCGCGGCCGCGGCCGCGGCCGCGGCCGCGGCCGCGGCCGCGGCCGCGGCCGCGGCCGCGGCCGCGGCCGCGGCCGCGGCCGCGGCCGCGGCCGCGGCCGCGGCCGCGGCCGCGGCCGCGGCCGCGGCCGCGGCCGCGGCCGCGGCCGCGGCCGCGGCCGCGGCCGCGGCCGCGGCCGCGGCCGCGGCCGCGGCCGCGGCCGCGGCCGCGGCCGCGGCCGCGGCCGCGGCCGCGGCCGCGGCCGCGGCCGCGGCCGCGGCCGCGGCCGCGGCCGCGGCCGCGGCCGCGGCCGCGGCCGCGGCCGCGGCCGCGGCCGCGGCCGCGGCCGCGGCCGCGGCCGCGGCCGCGGCCGCGGCCGCGGCCGCGGCCGCGGCCGCGGCCGCGGCCGCGGCCGCGGCCGCGGCCGCGGCCGCGGCCGCGGCCGCGGCCGCGGCCGCGGCCGCGGCCGCGGCCGCGGCCGCGGCCGCGGCCGCGGCCGCGGCCGCGGCCGCGGCCGCGGCCGCGGCCGCGGCCGCGGCCGCGGCCGCGGCCGCGGCCGCGGCCGCGGCCGCGGCCGCGGCCGCGGCCGCGGCCGCGGCCGCGGCCGCGGCCGCGGCCGCGGCCGCGGCCGCGGCCGCGGCCGCGGCCGCGGCCGCGGCCGCGGCCGCGGCCGCGGCCGCGGCCGCGGCCGCGGCCGCGGCCGCGGCCGCGGCCGCGGCCGCGGCCGCGGCCGCGGCCGCGGCCGCGGCCGCGGCCGCGGCCGCGGCCGCGGCCGCGGCCGCGGCCGCGGCCGCGGCCGCGGCCGCGGCCGCGGCCGCGGCCGCGGCCGCGGCCGCGGCCGCGGCCGCGGCCGCGGCCGCGGCCGCGGCCGCGGCCGCGGCCGCGGCCGCGGCCGCGGCCGCGGCCGCGGCCGCGGCCGCGGCCGCGGCCGCGGCCGCGGCCGCGGCCGCGGCCGCGGCCGCGGCCGCGGCCGCGGCCGCGGCCGCGGCCGCGGCCGCGGCCGCGGCCGCGGCCGCGGCCGCGGCCGCGGCCGCGGCCGCGGCCGCGGCCGCGGCCGCGGCCGCGGCCGCGGCCGCGGCCGCGGCCGCGGCCGCGGCCGCGGCCGCGGCCGCGGCCGCGGCCGCGGCCGCGGCCGCGGCCGCGGCCGCGGCCGCGGCCGCGGCCGCGGCCGCGGCCGCGGCCGCGGCCGCGGCCGCGGCCGCGGCCGCGGCCGCGGCCGCGGCCGCGGCCGCGGCCGCGGCCGCGGCCGCGGCCGCGGCCGCGGCCGCGGCCGCGGCCGCGGCCGCGGCCGCGGCCGCGGCCGCGGCCGCGGCCGCGGCCGCGGCCGCGGCCGCGGCCGCGGCCGCGGCCGCGGCCGCGGCCGCGGCCGCGGCCGCGGCCGCGGCCGCGGCCGCGGCCGCGGCCGCGGCCGCGGCCGCGGCCGCGGCCGCGGCCGCGGCCGCGGCCGCGGCCGCGGCCGCGGCCGCGGCCGCGGCCGCGGCCGCGGCCGCGGCCGCGGCCGCGGCCGCGGCCGCGGCCGCGGCCGCGGCCGCGGCCGCGGCCGCGGCCGCGGCCGCGGCCGCGGCCGCGGCCGCGGCCGCGGCCGCGGCCGCGGCCGCGGCCGCGGCCGCGGCCGCGGCCGCGGCCGCGGCCGCGGCCGCGGCCGCGGCCGCGGCCGCGGCCGCGGCCGCGGCCGCGGCCGCGGCCGCGGCCGCGGCCGCGGCCGCGGCCGCGGCCGCGGCCGCGGCCGCGGCCGCGGCCGCGGCCGCGGCCGCGGCCGCGGCCGCGGCCGCGGCCGCGGCCGCGGCCGCGGCCGCGGCCGCGGCCGCGGCCGCGGCCGCGGCCGCGGCCGCGGCCGCGGCCGCGGCCGCGGCCGCGGCCGCGGCCGCGGCCGCGGCCGCGGCCGCGGCCGCGGCCGCGGCCGCGGCCGCGGCCGCGGCCGCGGCCGCGGCCGCGGCCGCGGCCGCGGCCGCGGCCGCGGCCGCGGCCGCGGCCGCGGCCGCGGCCGCGGCCGCGGCCGCGGCCGCGGCCGCGGCCGCGGCCGCGGCCGCGGCCGCGGCCGCGGCCGCGGCCGCGGCCGCGGCCGCGGCCGCGGCCGCGGCCGCGGCCGCGGCCGCGGCCGCGGCCGCGGCCGCGGCCGCGGCCGCGGCCGCGGCCGCGGCCGCGGCCGCGGCCGCGGCCGCGGCCGCGGCCGCGGCCGCGGCCGCGGCCGCGGCCGCGGCCGCGGCCGCGGCCGCGGCCGCGGCCGCGGCCGCGGCCGCGGCCGCGGCCGCGGCCGCGGCCGCGGCCGCGGCCGCGGCCGCGGCCGCGGCCGCGGCCGCGGCCGCGGCCGCGGCCGCGGCCGCGGCCGCGGCCGCGGCCGCGGCCGCGGCCGCGGCCGCGGCCGCGGCCGCGGCCGCGGCCGCGGCCGCGGCCGCGGCCGCGGCCGCGGCCGCGGCCGCGGCCGCGGCCGCGGCCGCGGCCGCGGCCGCGGCCGCGGCCGCGGCCGCGGCCGCGGCCGCGGCCGCGGCCGCGGCCGCGGCCGCGGCCGCGGCCGCGGCCGCGGCCGCGGCCGCGGCCGCGGCCGCGGCCGCGGCCGCGGCCGCGGCCGCGGCCGCGGCCGCGGCCGCGGCCGCGGCCGCGGCCGCGGCCGCGGCCGCGGCCGCGGCCGCGGCCGCGGCCGCGGCCGCGGCCGCGGCCGCGGCCGCGGCCGCGGCCGCGGCCGCGGCCGCGGCCGCGGCCGCGGCCGCGGCCGCGGCCGCGGCCGCGGCCGCGGCCGCGGCCGCGGCCGCGGCCGCGGCCGCGGCCGCGGCCGCGGCCGCGGCCGCGGCCGCGGCCGCGGCCGCGGCCGCGGCCGCGGCCGCGGCCGCGGCCGCGGCCGCGGCCGCGGCCGCGGCCGCGGCCGCGGCCGCGGCCGCGGCCGCGGCCGCGGCCGCGGCCGCGGCCGCGGCCGCGGCCGCGGCCGCGGCCGCGGCCGCGGCCGCGGCCGCGGCCGCGGCCGCGGCCGCGGCCGCGGCCGCGGCCGCGGCCGCGGCCGCGGCCGCGGCCGCGGCCGCGGCCGCGGCCGCGGCCGCGGCCGCGGCCGCGGCCGCGGCCGCGGCCGCGGCCGCGGCCGCGGCCGCGGCCGCGGCCGCGGCCGCGGCCGCGGCCGCGGCCGCGGCCGCGGCCGCGGCCGCGGCCGCGGCCGCGGCCGCGGCCGCGGCCGCGGCCGCGGCCGCGGCCGCGGCCGCGGCCGCGGCCGCGGCCGCGGCCGCGGCCGCGGCCGCGGCCGCGGCCGCGGCCGCGGCCGCGGCCGCGGCCGCGGCCGCGGCCGCGGCCGCGGCCGCGGCCGCGGCCGCGGCCGCGGCCGCGGCCGCGGCCGCGGCCGCGGCCGCGGCCGCGGCCGCGGCCGCGGCCGCGGCCGCGGCCGCGGCCGCGGCCGCGGCCGCGGCCGCGGCCGCGGCCGCGGCCGCGGCCGCGGCCGCGGCCGCGGCCGCGGCCGCGGCCGCGGCCGCGGCCGCGGCCGCGGCCGCGGCCGCGGCCGCGGCCGCGGCCGCGGCCGCGGCCGCGGCCGCGGCCGCGGCCGCGGCCGCGGCCGCGGCCGCGGCCGCGGCCGCGGCCGCGGCCGCGGCCGCGGCCGCGGCCGCGGCCGCGGCCGCGGCCGCGGCCGCGGCCGCGGCCGCGGCCGCGGCCGCGGCCGCGGCCGCGGCCGCGGCCGCGGCCGCGGCCGCGGCCGCGGCCGCGGCCGCGGCCGCGGCCGCGGCCGCGGCCGCGGCCGCGGCCGCGGCCGCGGCCGCGGCCGCGGCCGCGGCCGCGGCCGCGGCCGCGGCCGCGGCCGCGGCCGCGGCCGCGGCCGCGGCCGCGGCCGCGGCCGCGGCCGCGGCCGCGGCCGCGGCCGCGGCCGCGGCCGCGGCCGCGGCCGCGGCCGCGGCCGCGGCCGCGGCCGCGGCCGCGGCCGCGGCCGCGGCCGCGGCCGCGGCCGCGGCCGCGGCCGCGGCCGCGGCCGCGGCCGCGGCCGCGGCCGCGGCCGCGGCCGCGGCCGCGGCCGCGGCCGCGGCCGCGGCCGCGGCCGCGGCCGCGGCCGCGGCCGCGGCCGCGGCCGCGGCCGCGGCCGCGGCCGCGGCCGCGGCCGCGGCCGCGGCCGCGGCCGCGGCCGCGGCCGCGGCCGCGGCCGCGGCCGCGGCCGCGGCCGCGGCCGCGGCCGCGGCCGCGGCCGCGGCCGCGGCCGCGGCCGCGGCCGCGGCCGCGGCCGCGGCCGCGGCCGCGGCCGCGGCCGCGGCCGCGGCCGCGGCCGCGGCCGCGGCCGCGGCCGCGGCCGCGGCCGCGGCCGCGGCCGCGGCCGCGGCCGCGGCCGCGGCCGCGGCCGCGGCCGCGGCCGCGGCCGCGGCCGCGGCCGCGGCCGCGGCCGCGGCCGCGGCCGCGGCCGCGGCCGCGGCCGCGGCCGCGGCCGCGGCCGCGGCCGCGGCCGCGGCCGCGGCCGCGGCCGCGGCCGCGGCCGCGGCCGCGGCCGCGGCCGCGGCCGCGGCCGCGGCCGCGGCCGCGGCCGCGGCCGCGGCCGCGGCCGCGGCCGCGGCCGCGGCCGCGGCCGCGGCCGCGGCCGCGGCCGCGGCCGCGGCCGCGGCCGCGGCCGCGGCCGCGGCCGCGGCCGCGGCCGCGGCCGCGGCCGCGGCCGCGGCCGCGGCCGCGGCCGCGGCCGCGGCCGCGGCCGCGGCCGCGGCCGCGGCCGCGGCCGCGGCCGCGGCCGCGGCCGCGGCCGCGGCCGCGGCCGCGGCCGCGGCCGCGGCCGCGGCCGCGGCCGCGGCCGCGGCCGCGGCCGCGGCCGCGGCCGCGGCCGCGGCCGCGGCCGCGGCCGCGGCCGCGGCCGCGGCCGCGGCCGCGGCCGCGGCCGCGGCCGCGGCCGCGGCCGCGGCCGCGGCCGCGGCCGCGGCCGCGGCCGCGGCCGCGGCCGCGGCCGCGGCCGCGGCCGCGGCCGCGGCCGCGGCCGCGGCCGCGGCCGCGGCCGCGGCCGCGGCCGCGGCCGCGGCCGCGGCCGCGGCCGCGGCCGCGGCCGCGGCCGCGGCCGCGGCCGCGGCCGCGGCCGCGGCCGCGGCCGCGGCCGCGGCCGCGGCCGCGGCCGCGGCCGCGGCCGCGGCCGCGGCCGCGGCCGCGGCCGCGGCCGCGGCCGCGGCCGCGGCCGCGGCCGCGGCCGCGGCCGCGGCCGCGGCCGCGGCCGCGGCCGCGGCCGCGGCCGCGGCCGCGGCCGCGGCCGCGGCCGCGGCCGCGGCCGCGGCCGCGGCCGCGGCCGCGGCCGCGGCCGCGGCCGCGGCCGCGGCCGCGGCCGCGGCCGCGGCCGCGGCCGCGGCCGCGGCCGCGGCCGCGGCCGCGGCCGCGGCCGCGGCCGCGGCCGCGGCCGCGGCCGCGGCCGCGGCCGCGGCCGCGGCCGCGGCCGCGGCCGCGGCCGCGGCCGCGGCCGCGGCCGCGGCCGCGGCCGCGGCCGCGGCCGCGGCCGCGGCCGCGGCCGCGGCCGCGGCCGCGGCCGCGGCCGCGGCCGCGGCCGCGGCCGCGGCCGCGGCCGCGGCCGCGGCCGCGGCCGCGGCCGCGGCCGCGGCCGCGGCCGCGGCCGCGGCCGCGGCCGCGGCCGCGGCCGCGGCCGCGGCCGCGGCCGCGGCCGCGGCCGCGGCCGCGGCCGCGGCCGCGGCCGCGGCCGCGGCCGCGGCCGCGGCCGCGGCCGCGGCCGCGGCCGCGGCCGCGGCCGCGGCCGCGGCCGCGGCCGCGGCCGCGGCCGCGGCCGCGGCCGCGGCCGCGGCCGCGGCCGCGGCCGCGGCCGCGGCCGCGGCCGCGGCCGCGGCCGCGGCCGCGGCCGCGGCCGCGGCCGCGGCCGCGGCCGCGGCCGCGGCCGCGGCCGCGGCCGCGGCCGCGGCCGCGGCCGCGGCCGCGGCCGCGGCCGCGGCCGCGGCCGCGGCCGCGGCCGCGGCCGCGGCCGCGGCCGCGGCCGCGGCCGCGGCCGCGGCCGCGGCCGCGGCCGCGGCCGCGGCCGCGGCCGCGGCCGCGGCCGCGGCCGCGGCCGCGGCCGCGGCCGCGGCCGCGGCCGCGGCCGCGGCCGCGGCCGCGGCCGCGGCCGCGGCCGCGGCCGCGGCCGCGGCCGCGGCCGCGGCCGCGGCCGCGGCCGCGGCCGCGGCCGCGGCCGCGGCCGCGGCCGCGGCCGCGGCCGCGGCCGCGGCCGCGGCCGCGGCCGCGGCCGCGGCCGCGGCCGCGGCCGCGGCCGCGGCCGCGGCCGCGGCCGCGGCCGCGGCCGCGGCCGCGGCCGCGGCCGCGGCCGCGGCCGCGGCCGCGGCCGCGGCCGCGGCCGCGGCCGCGGCCGCGGCCGCGGCCGCGGCCGCGGCCGCGGCCGCGGCCGCGGCCGCGGCCGCGGCCGCGGCCGCGGCCGCGGCCGCGGCCGCGGCCGCGGCCGCGGCCGCGGCCGCGGCCGCGGCCGCGGCCGCGGCCGCGGCCGCGGCCGCGGCCGCGGCCGCGGCCGCGGCCGCGGCCGCGGCCGCGGCCGCGGCCGCGGCCGCGGCCGCGGCCGCGGCCGCGGCCGCGGCCGCGGCCGCGGCCGCGGCCGCGGCCGCGGCCGCGGCCGCGGCCGCGGCCGCGGCCGCGGCCGCGGCCGCGGCCGCGGCCGCGGCCGCGGCCGCGGCCGCGGCCGCGGCCGCGGCCGCGGCCGCGGCCGCGGCCGCGGCCGCGGCCGCGGCCGCGGCCGCGGCCGCGGCCGCGGCCGCGGCCGCGGCCGCGGCCGCGGCCGCGGCCGCGGCCGCGGCCGCGGCCGCGGCCGCGGCCGCGGCCGCGGCCGCGGCCGCGGCCGCGGCCGCGGCCGCGGCCGCGGCCGCGGCCGCGGCCGCGGCCGCGGCCGCGGCCGCGGCCGCGGCCGCGGCCGCGGCCGCGGCCGCGGCCGCGGCCGCGGCCGCGGCCGCGGCCGCGGCCGCGGCCGCGGCCGCGGCCGCGGCCGCGGCCGCGGCCGCGGCCGCGGCCGCGGCCGCGGCCGCGGCCGCGGCCGCGGCCGCGGCCGCGGCCGCGGCCGCGGCCGCGGCCGCGGCCGCGGCCGCGGCCGCGGCCGCGGCCGCGGCCGCGGCCGCGGCCGCGGCCGCGGCCGCGGCCGCGGCCGCGGCCGCGGCCGCGGCCGCGGCCGCGGCCGCGGCCGCGGCCGCGGCCGCGGCCGCGGCCGCGGCCGCGGCCGCGGCCGCGGCCGCGGCCGCGGCCGCGGCCGCGGCCGCGGCCGCGGCCGCGGCCGCGGCCGCGGCCGCGGCCGCGGCCGCGGCCGCGGCCGCGGCCGCGGCCGCGGCCGCGGCCGCGGCCGCGGCCGCGGCCGCGGCCGCGGCCGCGGCCGCGGCCGCGGCCGCGGCCGCGGCCGCGGCCGCGGCCGCGGCCGCGGCCGCGGCCGCGGCCGCGGCCGCGGCCGCGGCCGCGGCCGCGGCCGCGGCCGCGGCCGCGGCCGCGGCCGCGGCCGCGGCCGCGGCCGCGGCCGCGGCCGCGGCCGCGGCCGCGGCCGCGGCCGCGGCCGCGGCCGCGGCCGCGGCCGCGGCCGCGGCCGCGGCCGCGGCCGCGGCCGCGGCCGCGGCCGCGGCCGCGGCCGCGGCCGCGGCCGCGGCCGCGGCCGCGGCCGCGGCCGCGGCCGCGGCCGCGGCCGCGGCCGCGGCCGCGGCCGCGGCCGCGGCCGCGGCCGCGGCCGCGGCCGCGGCCGCGGCCGCGGCCGCGGCCGCGGCCGCGGCCGCGGCCGCGGCCGCGGCCGCGGCCGCGGCCGCGGCCGCGGCCGCGGCCGCGGCCGCGGCCGCGGCCGCGGCCGCGGCCGCGGCCGCGGCCGCGGCCGCGGCCGCGGCCGCGGCCGCGGCCGCGGCCGCGGCCGCGGCCGCGGCCGCGGCCGCGGCCGCGGCCGCGGCCGCGGCCGCGGCCGCGGCCGCGGCCGCGGCCGCGGCCGCGGCCGCGGCCGCGGCCGCGGCCGCGGCCGCGGCCGCGGCCGCGGCCGCGGCCGCGGCCGCGGCCGCGGCCGCGGCCGCGGCCGCGGCCGCGGCCGCGGCCGCGGCCGCGGCCGCGGCCGCGGCCGCGGCCGCGGCCGCGGCCGCGGCCGCGGCCGCGGCCGCGGCCGCGGCCGCGGCCGCGGCCGCGGCCGCGGCCGCGGCCGCGGCCGCGGCCGCGGCCGCGGCCGCGGCCGCGGCCGCGGCCGCGGCCGCGGCCGCGGCCGCGGCCGCGGCCGCGGCCGCGGCCGCGGCCGCGGCCGCGGCCGCGGCCGCGGCCGCGGCCGCGGCCGCGGCCGCGGCCGCGGCCGCGGCCGCGGCCGCGGCCGCGGCCGCGGCCGCGGCCGCGGCCGCGGCCGCGGCCGCGGCCGCGGCCGCGGCCGCGGCCGCGGCCGCGGCCGCGGCCGCGGCCGCGGCCGCGGCCGCGGCCGCGGCCGCGGCCGCGGCCGCGGCCGCGGCCGCGGCCGCGGCCGCGGCCGCGGCCGCGGCCGCGGCCGCGGCCGCGGCCGCGGCCGCGGCCGCGGCCGCGGCCGCGGCCGCGGCCGCGGCCGCGGCCGCGGCCGCGGCCGCGGCCGCGGCCGCGGCCGCGGCCGCGGCCGCGGCCGCGGCCGCGGCCGCGGCCGCGGCCGCGGCCGCGGCCGCGGCCGCGGCCGCGGCCGCGGCCGCGGCCGCGGCCGCGGCCGCGGCCGCGGCCGCGGCCGCGGCCGCGGCCGCGGCCGCGGCCGCGGCCGCGGCCGCGGCCGCGGCCGCGGCCGCGGCCGCGGCCGCGGCCGCGGCCGCGGCCGCGGCCGCGGCCGCGGCCGCGGCCGCGGCCGCGGCCGCGGCCGCGGCCGCGGCCGCGGCCGCGGCCGCGGCCGCGGCCGCGGCCGCGGCCGCGGCCGCGGCCGCGGCCGCGGCCGCGGCCGCGGCCGCGGCCGCGGCCGCGGCCGCGGCCGCGGCCGCGGCCGCGGCCGCGGCCGCGGCCGCGGCCGCGGCCGCGGCCGCGGCCGCGGCCGCGGCCGCGGCCGCGGCCGCGGCCGCGGCCGCGGCCGCGGCCGCGGCCGCGGCCGCGGCCGCGGCCGCGGCCGCGGCCGCGGCCGCGGCCGCGGCCGCGGCCGCGGCCGCGGCCGCGGCCGCGGCCGCGGCCGCGGCCGCGGCCGCGGCCGCGGCCGCGGCCGCGGCCGCGGCCGCGGCCGCGGCCGCGGCCGCGGCCGCGGCCGCGGCCGCGGCCGCGGCCGCGGCCGCGGCCGCGGCCGCGGCCGCGGCCGCGGCCGCGGCCGCGGCCGCGGCCGCGGCCGCGGCCGCGGCCGCGGCCGCGGCCGCGGCCGCGGCCGCGGCCGCGGCCGCGGCCGCGGCCGCGGCCGCGGCCGCGGCCGCGGCCGCGGCCGCGGCCGCGGCCGCGGCCGCGGCCGCGGCCGCGGCCGCGGCCGCGGCCGCGGCCGCGGCCGCGGCCGCGGCCGCGGCCGCGGCCGCGGCCGCGGCCGCGGCCGCGGCCGCGGCCGCGGCCGCGGCCGCGGCCGCGGCCGCGGCCGCGGCCGCGGCCGCGGCCGCGGCCGCGGCCGCGGCCGCGGCCGCGGCCGCGGCCGCGGCCGCGGCCGCGGCCGCGGCCGCGGCCGCGGCCGCGGCCGCGGCCGCGGCCGCGGCCGCGGCCGCGGCCGCGGCCGCGGCCGCGGCCGCGGCCGCGGCCGCGGCCGCGGCCGCGGCCGCGGCCGCGGCCGCGGCCGCGGCCGCGGCCGCGGCCGCGGCCGCGGCCGCGGCCGCGGCCGCGGCCGCGGCCGCGGCCGCGGCCGCGGCCGCGGCCGCGGCCGCGGCCGCGGCCGCGGCCGCGGCCGCGGCCGCGGCCGCGGCCGCGGCCGCGGCCGCGGCCGCGGCCGCGGCCGCGGCCGCGGCCGCGGCCGCGGCCGCGGCCGCGGCCGCGGCCGCGGCCGCGGCCGCGGCCGCGGCCGCGGCCGCGGCCGCGGCCGCGGCCGCGGCCGCGGCCGCGGCCGCGGCCGCGGCCGCGGCCGCGGCCGCGGCCGCGGCCGCGGCCGCGGCCGCGGCCGCGGCCGCGGCCGCGGCCGCGGCCGCGGCCGCGGCCGCGGCCGCGGCCGCGGCCGCGGCCGCGGCCGCGGCCGCGGCCGCGGCCGCGGCCGCGGCCGCGGCCGCGGCCGCGGCCGCGGCCGCGGCCGCGGCCGCGGCCGCGGCCGCGGCCGCGGCCGCGGCCGCGGCCGCGGCCGCGGCCGCGGCCGCGGCCGCGGCCGCGGCCGCGGCCGCGGCCGCGGCCGCGGCCGCGGCCGCGGCCGCGGCCGCGGCCGCGGCCGCGGCCGCGGCCGCGGCCGCGGCCGCGGCCGCGGCCGCGGCCGCGGCCGCGGCCGCGGCCGCGGCCGCGGCCGCGGCCGCGGCCGCGGCCGCGGCCGCGGCCGCGGCCGCGGCCGCGGCCGCGGCCGCGGCCGCGGCCGCGGCCGCGGCCGCGGCCGCGGCCGCGGCCGCGGCCGCGGCCGCGGCCGCGGCCGCGGCCGCGGCCGCGGCCGCGGCCGCGGCCGCGGCCGCGGCCGCGGCCGCGGCCGCGGCCGCGGCCGCGGCCGCGGCCGCGGCCGCGGCCGCGGCCGCGGCCGCGGCCGCGGCCGCGGCCGCGGCCGCGGCCGCGGCCGCGGCCGCGGCCGCGGCCGCGGCCGCGGCCGCGGCCGCGGCCGCGGCCGCGGCCGCGGCCGCGGCCGCGGCCGCGGCCGCGGCCGCGGCCGCGGCCGCGGCCGCGGCCGCGGCCGCGGCCGCGGCCGCGGCCGCGGCCGCGGCCGCGGCCGCGGCCGCGGCCGCGGCCGCGGCCGCGGCCGCGGCCGCGGCCGCGGCCGCGGCCGCGGCCGCGGCCGCGGCCGCGGCCGCGGCCGCGGCCGCGGCCGCGGCCGCGGCCGCGGCCGCGGCCGCGGCCGCGGCCGCGGCCGCGGCCGCGGCCGCGGCCGCGGCCGCGGCCGCGGCCGCGGCCGCGGCCGCGGCCGCGGCCGCGGCCGCGGCCGCGGCCGCGGCCGCGGCCGCGGCCGCGGCCGCGGCCGCGGCCGCGGCCGCGGCCGCGGCCGCGGCCGCGGCCGCGGCCGCGGCCGCGGCCGCGGCCGCGGCCGCGGCCGCGGCCGCGGCCGCGGCCGCGGCCGCGGCCGCGGCCGCGGCCGCGGCCGCGGCCGCGGCCGCGGCCGCGGCCGCGGCCGCGGCCGCGGCCGCGGCCGCGGCCGCGGCCGCGGCCGCGGCCGCGGCCGCGGCCGCGGCCGCGGCCGCGGCCGCGGCCGCGGCCGCGGCCGCGGCCGCGGCCGCGGCCGCGGCCGCGGCCGCGGCCGCGGCCGCGGCCGCGGCCGCGGCCGCGGCCGCGGCCGCGGCCGCGGCCGCGGCCGCGGCCGCGGCCGCGGCCGCGGCCGCGGCCGCGGCCGCGGCCGCGGCCGCGGCCGCGGCCGCGGCCGCGGCCGCGGCCGCGGCCGCGGCCGCGGCCGCGGCCGCGGCCGCGGCCGCGGCCGCGGCCGCGGCCGCGGCCGCGGCCGCGGCCGCGGCCGCGGCCGCGGCCGCGGCCGCGGCCGCGGCCGCGGCCGCGGCCGCGGCCGCGGCCGCGGCCGCGGCCGCGGCCGCGGCCGCGGCCGCGGCCGCGGCCGCGGCCGCGGCCGCGGCCGCGGCCGCGGCCGCGGCCGCGGCCGCGGCCGCGGCCGCGGCCGCGGCCGCGGCCGCGGCCGCGGCCGCGGCCGCGGCCGCGGCCGCGGCCGCGGCCGCGGCCGCGGCCGCGGCCGCGGCCGCGGCCGCGGCCGCGGCCGCGGCCGCGGCCGCGGCCGCGGCCGCGGCCGCGGCCGCGGCCGCGGCCGCGGCCGCGGCCGCGGCCGCGGCCGCGGCCGCGGCCGCGGCCGCGGCCGCGGCCGCGGCCGCGGCCGCGGCCGCGGCCGCGGCCGCGGCCGCGGCCGCGGCCGCGGCCGCGGCCGCGGCCGCGGCCGCGGCCGCGGCCGCGGCCGCGGCCGCGGCCGCGGCCGCGGCCGCGGCCGCGGCCGCGGCCGCGGCCGCGGCCGCGGCCGCGGCCGCGGCCGCGGCCGCGGCCGCGGCCGCGGCCGCGGCCGCGGCCGCGGCCGCGGCCGCGGCCGCGGCCGCGGCCGCGGCCGCGGCCGCGGCCGCGGCCGCGGCCGCGGCCGCGGCCGCGGCCGCGGCCGCGGCCGCGGCCGCGGCCGCGGCCGCGGCCGCGGCCGCGGCCGCGGCCGCGGCCGCGGCCGCGGCCGCGGCCGCGGCCGCGGCCGCGGCCGCGGCCGCGGCCGCGGCCGCGGCCGCGGCCGCGGCCGCGGCCGCGGCCGCGGCCGCGGCCGCGGCCGCGGCCGCGGCCGCGGCCGCGGCCGCGGCCGCGGCCGCGGCCGCGGCCGCGGCCGCGGCCGCGGCCGCGGCCGCGGCCGCGGCCGCGGCCGCGGCCGCGGCCGCGGCCGCGGCCGCGGCCGCGGCCGCGGCCGCGGCCGCGGCCGCGGCCGCGGCCGCGGCCGCGGCCGCGGCCGCGGCCGCGGCCGCGGCCGCGGCCGCGGCCGCGGCCGCGGCCGCGGCCGCGGCCGCGGCCGCGGCCGCGGCCGCGGCCGCGGCCGCGGCCGCGGCCGCGGCCGCGGCCGCGGCCGCGGCCGCGG

**Table S2. Summary of primers for rat RT‑qPCR**

|  | forward primer 5′-3′ | reverse primer 5′3′ |
| --- | --- | --- |
| FoxO3 | AAACGGCTCACTTTGTCCCAGATC | CCTCGGCTCTTGGTGTACTTGTTG |
| Aggrecan（Acan） | GGCTGTCCTGGTGATAGAGG | CTGCTGCTGCTGTTGTTGGT |
| Col2a1 | GATGGCTGCACGAAACATACC | GCCCTATGTCCACACCGAAT |
| ADAMTS5 | GCCCACCTAACGGCAAATCT | AGGACACCTGCGTATTTGGG |
| MMP13 | GTTGATAGACTCCGAGAAATGC | GTTAAGTTTGTTTGGGACCATT |
| GAPDH | CGACTTCAACAGCAACTCCCACTCTTCC | TGGGTGGTCCAGGGTTTCTTACTCCTT |
